# Supplementary material for: Identification of upstream transcription factor binding sites in orthologous genes using mixed Student’s t-test statistics
Source: PLoS Comput Biol. 2022 Jun 7;18(6):e1009773. doi: 10.1371/journal.pcbi.1009773 (PMC9205514; doi:10.1371/journal.pcbi.1009773)
Supplement: S1 Text — (DOCX) [file pcbi.1009773.s001.docx]

S1_Text. Proof of the mixed Students’ *t*-test

(1) One-sample test of whether the mean of a population is statically different from a known or hypothesized value.

$$t^{1}=\frac{\bar{X}-\mu}{\sqrt{\frac{\sum_{i=1}^{n} {(x_{i}-\bar{x})}^{2}}{n}}} (1)$$

(2) Two-sample test of whether the means of two populations are statically equal.

$$t^{2}=\frac{\bar{X}_{1}-\bar{X}_{2}}{\sqrt{\frac{\left( n_{1}-1 \right)s_{1}^{2}+\left( n_{2}-1 \right)s_{2}^{2}}{n_{1}+n_{2}-2}}\cdot\sqrt{\frac{1}{n_{1}}+\frac{1}{n_{2}}}} (2)$$

(3) The mixed Student’s *t*-test statistic can be calculated by combining the one-sample Student’s *t*-test and independent two-sample Student’s *t*-test together.

In this study, a similar but different statistical problem to Student’s *t*-test was proposed: give a background set (*bkg*) and testing set (*obs*), see if one value (*one*) from the *obs* is significantly different from the mean of the values in *bkg*.

$$t^{'}=\frac{\bar{X}_{bkg}-one}{\sqrt{\frac{\left( n_{obs}-1 \right)s_{obs}^{2}+\left( n_{bkg}-1 \right)s_{bkg}^{2}}{n_{obs}+n_{bkg}-2}}\cdot\sqrt{\frac{1}{n_{obs}}+\frac{1}{n_{bkg}}}} (3)$$

$$df=\frac{\left( \frac{s_{obs}^{2}}{n_{obs}}+\frac{s_{bkg}^{2}}{n_{bkg}} \right)^{2}}{\frac{\left( {s_{obs}^{2}}/{n_{obs}} \right)^{2}}{n_{obs}-1}+\frac{\left( {s_{bkg}^{2}}/{n_{bkg}} \right)^{2}}{n_{bkg}-1}} (4)$$

If distributions of *obs* and *bkg* were the same, *f_t’_(n_obs_, n_bkg_)* is continues at *(n_all_, n_all_)* when *n_all_* > 2, whereas *n_all_* = *n_obs_* + *n_bkg_*_._

$$\bar{X}_{bkg}=\bar{X}_{obs}=\bar{X}_{all}$$

$$t^{'}=\frac{\bar{X}_{bkg}-one}{\sqrt{\frac{\left( n_{obs}-1 \right)s_{obs}^{2}+\left( n_{bkg}-1 \right)s_{bkg}^{2}}{n_{obs}+n_{bkg}-2}}\cdot\sqrt{\frac{1}{n_{obs}}+\frac{1}{n_{bkg}}}}$$

$$\lim_{(n_{obs},n_{bkg})\to(n_{all/2},n_{all/2})} t'=\frac{\bar{X}_{bkg}-one}{\sqrt{\frac{\left( \frac{n_{all}}{2}-1 \right)\frac{s_{all}^{2}}{4}+\left( \frac{n_{all}}{2}-1 \right)\frac{s_{all}^{2}}{4}}{n_{all}-2}}\cdot\sqrt{\frac{4}{n_{all}}}}$$

$$=\frac{\bar{X}_{bkg}-one}{\sqrt{\frac{s_{all}^{2}}{n_{all}}}}$$

Which is equivalent to Equation 1.

If observation *one* located at the mean of the *obs*,

$$\bar{X}_{bkg}=\bar{X}_{obs}=one$$

$$t^{'}=\frac{\left( \bar{X}_{bkg}-one \right)}{\sqrt{\frac{\left( n_{obs}-1 \right)s_{obs}^{2}+\left( n_{obs}-1 \right)s_{bkg}^{2}}{n_{obs}+n_{bkg}-2}}\cdot\sqrt{\frac{1}{n_{obs}}+\frac{1}{n_{bkg}}}}$$

$$=\frac{\left( \bar{X}_{bkg}-\bar{X}_{obs} \right)}{\sqrt{\frac{\left( n_{obs}-1 \right)s_{obs}^{2}+\left( n_{bkg}-1 \right)s_{bkg}^{2}}{n_{obs}+n_{bkg}-2}}\cdot\sqrt{\frac{1}{n_{obs}}+\frac{1}{n_{bkg}}}}$$

Which is equivalent to Equation 2.
