# Supplementary material for: Identification of upstream transcription factor binding sites in orthologous genes using mixed Student’s t-test statistics
Source: PLoS Comput Biol. 2022 Jun 7;18(6):e1009773. doi: 10.1371/journal.pcbi.1009773 (PMC9205514; doi:10.1371/journal.pcbi.1009773)
Supplement: S1 Table — (DOCX) [file pcbi.1009773.s002.docx]

S1_Table. Detailed information for genomes in Ensembl Biomart web tool release 100

Table S1. Detailed information for genomes in Ensembl Biomart web tool release 100

| Index | Common name | Version | Scientific name |
| --- | --- | --- | --- |
| 1 | Pachon cavefish | Astyanax_mexicanus-2.0 | Astyanax mexicanus |
| 2 | Hybrid - Bos Taurus | UOA_Angus_1 | Bos indicus x Bos taurus |
| 3 | Hybrid - Bos Indicus | UOA_Angus_1 | Bos indicus x Bos taurus |
| 4 | Dog - Basenji | Basenji_breed-1.1 | Canis lupus familiaris |
| 5 | Dog - Great Dane | Basenji_breed-1.1 | Canis lupus familiaris |
| 6 | Capuchin | Cebus_imitator-1.0 | Cebus capucinus imitator |
| 7 | Rhinoceros (Pre) | - | Ceratotherium simum simum |
| 8 | C.savignyi | CSAV 2.0 | Ciona savignyi |
| 9 | Chinese hamster CHOK1GS | CHOK1GS_HDv1 | Cricetulus griseus |
| 10 | Chinese hamster CriGri | CHOK1GS_HDv1 | Cricetulus griseus |
| 11 | Chinese hamster PICR | CHOK1GS_HDv1 | Cricetulus griseus |
| 12 | Rufous-capped babbler | ASM869450v1 | Cyanoderma ruficeps |
| 13 | Common carp german mirror | German_Mirror_carp_1.0 | Cyprinus carpio |
| 14 | Common carp hebao red | German_Mirror_carp_1.0 | Cyprinus carpio |
| 15 | Common carp huanghe | German_Mirror_carp_1.0 | Cyprinus carpio |
| 16 | Hedgehog (Pre) | - | Erinaceus europaeus |
| 17 | Hedgehog | - | Erinaceus europaeus |
| 18 | Stickleback | BROAD S1 | Gasterosteus aculeatus |
| 19 | Gorilla | gorGor4 | Gorilla gorilla gorilla |
| 20 | Naked mole-rat male | HetGla_1.0 | Heterocephalus glaber |
| 21 | Naked mole-rat female | HetGla_1.0 | Heterocephalus glaber |
| 22 | Jewelled blenny | fSalaFa1.1 | Jewelled blenny |
| 23 | Elephant | Loxafr3.0 | Loxodonta africana |
| 24 | Mouse 129S1/SvImJ | GRCm38 | Mus musculus |
| 25 | Mouse A/J | GRCm38 | Mus musculus |
| 26 | Mouse AKR/J | GRCm38 | Mus musculus |
| 27 | Mouse BALB/cJ | GRCm38 | Mus musculus |
| 28 | Mouse C3H/HeJ | GRCm38 | Mus musculus |
| 29 | Mouse C57BL/6NJ | GRCm38 | Mus musculus |
| 30 | Mouse CBA/J | GRCm38 | Mus musculus |
| 31 | Mouse DBA/2J | GRCm38 | Mus musculus |
| 32 | Mouse FVB/NJ | GRCm38 | Mus musculus |
| 33 | Mouse LP/J | GRCm38 | Mus musculus |
| 34 | Mouse NOD/ShiLtJ | GRCm38 | Mus musculus |
| 35 | Mouse NZO/HlLtJ | GRCm38 | Mus musculus |
| 36 | Mouse CAST/EiJ | CAST_EiJ_v1 | Mus musculus castaneus |
| 37 | Mouse WSB/EiJ | WSB_EiJ_v1 | Mus musculus domesticus |
| 38 | Mouse PWK/PhJ | PWK_PhJ_v1 | Mus musculus musculus |
| 39 | Pika (Pre) | OchPri2.0-Ens | Ochotona princeps |
| 40 | Platypus | OANA5 | Ornithorhynchus anatinus |
| 41 | Aardvark (Pre) | - | Orycteropus afer afer |
| 42 | Japanese medaka HSOK | ASM223471v1 | Oryzias latipes |
| 43 | Japanese medaka HNI | ASM223471v1 | Oryzias latipes |
| 44 | Hamadryas Baboon (Pre) | - | Papio hamadryas |
| 45 | Amazon molly | Poecilia_formosa-5.1.2 | Poecilia formosa |
| 46 | Tasmanian devil | Devil_ref v7.0 | Sarcophilus harrisii |
| 47 | Shrew (Pre) | - | Sorex araneus |
| 48 | Shrew | - | Sorex araneus |
| 49 | Pig - Bamei | minipig_v1.0 | Sus scrofa |
| 50 | Pig - Berkshire | minipig_v1.0 | Sus scrofa |
| 51 | Pig - Hampshire | minipig_v1.0 | Sus scrofa |
| 52 | Pig - Jinhua | minipig_v1.0 | Sus scrofa |
| 53 | Pig - Landrace | minipig_v1.0 | Sus scrofa |
| 54 | Pig - Largewhite | minipig_v1.0 | Sus scrofa |
| 55 | Pig - Meishan | minipig_v1.0 | Sus scrofa |
| 56 | Pig - Wuzhishan | minipig_v1.0 | Sus scrofa |
| 57 | Pig - Pietrain | minipig_v1.0 | Sus scrofa |
| 58 | Pig - Rongchang | minipig_v1.0 | Sus scrofa |
| 59 | Pig - Tibetan | minipig_v1.0 | Sus scrofa |
| 60 | Pig USMARC | minipig_v1.0 | Sus scrofa |
| 61 | Pig FPC map (Pre) | - | Sus scrofa map |
| 62 | Tetraodon | TETRAODON 8.0 | Tetraodon nigroviridis |
| 63 | Tree Shrew | tupBel1 | Tupaia belangeri |
| 64 | Dolphin (Pre) | - | Tursiops truncatus |
| 65 | Indian medaka | Om_v0.7.RACA | Oryzias melastigma |
| 66 | Elephant shark | Callorhinchus_milii-6.1.3 | Callorhinchus milii |
| 67 | Painted turtle | Chrysemys_picta_bellii-3.0.3 | Chrysemys picta bellii |
| 68 | Atlantic herring | Ch_v2.0.2 | Clupea harengus |
| 69 | Bicolor damselfish | Stegastes_partitus-1.0.2 | Stegastes partitus |
| 70 | Mummichog | Fundulus_heteroclitus-3.0.2 | Fundulus heteroclitus |
| 71 | Red-bellied piranha | Pygocentrus_nattereri-1.0.2 | Pygocentrus nattereri |
| 72 | Blind barbel | SAMN03320099.WGS_v1.1 | Sinocyclocheilus anshuiensis |
| 73 | Golden-line barbel | SAMN03320097.WGS_v1.1 | Sinocyclocheilus grahami |
| 74 | Monterrey platyfish | Xiphophorus_couchianus-4.0.1 | Xiphophorus couchianus |
| 75 | Tarsier | Tarsius_syrichta-2.0.1 | Carlito syrichta |
| 76 | White-throated sparrow | Zonotrichia_albicollis-1.0.1 | Zonotrichia albicollis |
| 77 | Angola colobus | Cang.pa_1.0 | Colobus angolensis palliatus |
| 78 | Drill | Mleu.le_1.0 | Mandrillus leucophaeus |
| 79 | Upper Galilee mountains blind mole rat | S.galili_v1.0 | Nannospalax galili |
| 80 | American beaver | C.can_genome_v1.0 | Castor canadensis |
| 81 | American mink | NNQGG.v01 | Neovison vison |
| 82 | Mouse | GRCm38 | Mus musculus |
| 83 | Human | GRCh38 | Homo sapiens |
| 84 | Canada lynx | mLynCan4_v1.p | Lynx canadensis |
| 85 | Goodes thornscrub tortoise | rGopEvg1_v1.p | Gopherus evgoodei |
| 86 | Greater horseshoe bat | mRhiFer1_v1.p | Rhinolophus ferrumequinum |
| 87 | Kakapo | bStrHab1_v1.p | Strigops habroptila |
| 88 | Zebra finch | bTaeGut1_v1.p | Taeniopygia guttata |
| 89 | P. magnuspinnatus | PM.fa | Periophthalmus magnuspinnatus |
| 90 | Platyfish | X_maculatus-5.0-male | Xiphophorus maculatus |
| 91 | Pika | OchPri2.0-Ens | Ochotona princeps |
| 92 | Guppy | Guppy_female_1.0_MT | Poecilia reticulata |
| 93 | D. melanogaster | BDGP6.28 | Drosophila melanogaster |
| 94 | Flycatcher | FicAlb_1.4 | Ficedula albicollis |
| 95 | Budgerigar | Melopsittacus_undulatus_6.3 | Melopsittacus undulatus |
| 96 | Channel catfish | IpCoco_1.2 | Ictalurus punctatus |
| 97 | Cow | ARS-UCD1.2 | Bos taurus |
| 98 | Eastern happy | fAstCal1.2 | Astatotilapia calliptera |
| 99 | Fugu | fTakRub1.2 | Takifugu rubripes |
| 100 | Golden eagle | bAquChr1.2 | Aquila chrysaetos chrysaetos |
| 101 | Hagfish | Eburgeri_3.2 | Eptatretus burgeri |
| 102 | Red fox | VulVul2.2 | Vulpes vulpes |
| 103 | Siamese fighting fish | fBetSpl5.2 | Betta splendens |
| 104 | Alpine marmot | marMar2.1 | Marmota marmota marmota |
| 105 | Asian bonytongue | fSclFor1.1 | Scleropages formosus |
| 106 | Blunt-snouted clingfish | fGouWil2.1 | Gouania willdenowi |
| 107 | Bonobo | panpan1.1 | Pan paniscus |
| 108 | Central bearded dragon | pvi1.1 | Pogona vitticeps |
| 109 | Channel bull blenny | fCotGob3.1 | Cottoperca gobio |
| 110 | Climbing perch | fAnaTes1.1 | Anabas testudineus |
| 111 | Denticle herring | fDenClu1.1 | Denticeps clupeoides |
| 112 | Dog | Basenji_breed-1.1 | Canis lupus familiaris |
| 113 | Gilthead seabream | fSpaAur1.1 | Sparus aurata |
| 114 | Great Tit | Parus_major1.1 | Parus major |
| 115 | Horned golden-line barbel | SAMN03320098_v1.1 | Sinocyclocheilus rhinocerous |
| 116 | Indian glassy fish | fParRan2.1 | Parambassis ranga |
| 117 | Javanese ricefish | OJAV_1.1 | Oryzias javanicus |
| 118 | Koala | phaCin_unsw_v4.1 | Phascolarctos cinereus |
| 119 | Live sharksucker | fEcheNa1.1 | Echeneis naucrates |
| 120 | Northern American deer mouse | HU_Pman_2.1 | Peromyscus maniculatus bairdii |
| 121 | Orbiculate cardinalfish | fSphaOr1.1 | Sphaeramia orbicularis |
| 122 | P. kingsleyae | PKINGS_0.1 | Paramormyrops kingsleyae |
| 123 | Pig | minipig_v1.0 | Sus scrofa |
| 124 | Pinecone soldierfish | fMyrMur1.1 | Myripristis murdjan |
| 125 | Reedfish | fErpCal1.1 | Erpetoichthys calabaricus |
| 126 | River trout | fSalTru1.1 | Salmo trutta |
| 127 | Ryukyu mouse | CAROLI_EIJ_v1.1 | Mus caroli |
| 128 | Sheep | Oar_v3.1 | Ovis aries |
| 129 | Shrew mouse | PAHARI_EIJ_v1.1 | Mus pahari |
| 130 | Tropical clawed frog | Xenopus_tropicalis_v9.1 | Xenopus tropicalis |
| 131 | Turkey | Turkey_2.01 | Meleagris gallopavo |
| 132 | Vervet-AGM | ChlSab1.1 | Chlorocebus sabaeus |
| 133 | Zig-zag eel | fMasArm1.1 | Mastacembelus armatus |
| 134 | American bison | Bison_UMD1.0 | Bison bison bison |
| 135 | Anole lizard | AnoCar2.0 | Anolis carolinensis |
| 136 | Armadillo | Dasnov3.0 | Dasypus novemcinctus |
| 137 | Blue-crowned manakin | Lepidothrix_coronata-1.0 | Lepidothrix coronata |
| 138 | Blue-ringed sea krait | latLat_1.0 | Laticauda laticaudata |
| 139 | Bolivian squirrel monkey | SaiBol1.0 | Saimiri boliviensis boliviensis |
| 140 | Brazilian guinea pig | CavAp1.0 | Cavia aperea |
| 141 | Burton's mouthbrooder | AstBur1.0 | Haplochromis burtoni |
| 142 | Cat | Felis_catus_9.0 | Felis catus |
| 143 | Chimpanzee | Pan_tro_3.0 | Pan troglodytes |
| 144 | Chinese softshell turtle | PelSin_1.0 | Pelodiscus sinensis |
| 145 | Chinook salmon | Otsh_v1.0 | Oncorhynchus tshawytscha |
| 146 | Clown anemonefish | AmpOce1.0 | Amphiprion ocellaris |
| 147 | Common snapping turtle | Chelydra_serpentina-1.0 | Chelydra serpentina |
| 148 | Common wall lizard | PodMur_1.0 | Podarcis muralis |
| 149 | Coquerel's sifaka | Pcoq_1.0 | Propithecus coquereli |
| 150 | Crab-eating macaque | Macaca_fascicularis_5.0 | Macaca fascicularis |
| 151 | Damara mole rat | DMR_v1.0 | Fukomys damarensis |
| 152 | Degu | OctDeg1.0 | Octodon degus |
| 153 | Domestic yak | LU_Bosgru_v3.0 | Bos grunniens |
| 154 | Duck | CAU_duck1.0 | Anas platyrhynchos platyrhynchos |
| 155 | Eurasian sparrowhawk | Accipiter_nisus_ver1.0 | Accipiter nisus |
| 156 | European seabass | seabass_V1.0 | Dicentrarchus labrax |
| 157 | Ferret | MusPutFur1.0 | Mustela putorius furo |
| 158 | Gelada | Tgel_1.0 | Theropithecus gelada |
| 159 | Gibbon | Nleu_3.0 | Nomascus leucogenys |
| 160 | Golden Hamster | MesAur1.0 | Mesocricetus auratus |
| 161 | Golden pheasant | Chrysolophus_pictus_GenomeV1.0 | Chrysolophus pictus |
| 162 | Greater amberjack | Sdu_1.0 | Seriola dumerili |
| 163 | Greater bamboo lemur | Prosim_1.0 | Prolemur simus |
| 164 | Guinea Pig | Cavpor3.0 | Cavia porcellus |
| 165 | Helmeted guineafowl | NumMel1.0 | Numida meleagris |
| 166 | Horse | EquCab3.0 | Equus caballus |
| 167 | Indian peafowl | AIIM_Pcri_1.0 | Pavo cristatus |
| 168 | Japanese quail | Coturnix_japonica_2.0 | Coturnix japonica |
| 169 | Kangaroo rat | Dord_2.0 | Dipodomys ordii |
| 170 | Lamprey | Pmarinus_7.0 | Petromyzon marinus |
| 171 | Large yellow croaker | L_crocea_2.0 | Larimichthys crocea |
| 172 | Leopard | PanPar1.0 | Panthera pardus |
| 173 | Lesser Egyptian jerboa | JacJac1.0 | Jaculus jaculus |
| 174 | Lion | PanLeo1.0 | Panthera leo |
| 175 | Long-tailed chinchilla | ChiLan1.0 | Chinchilla lanigera |
| 176 | Lyretail cichlid | NeoBri1.0 | Neolamprologus brichardi |
| 177 | Ma's night monkey | Anan_2.0 | Aotus nancymaae |
| 178 | Makobe Island cichlid | PunNye1.0 | Pundamilia nyererei |
| 179 | Medium ground-finch | GeoFor_1.0 | Geospiza fortis |
| 180 | Mexican tetra | Astyanax_mexicanus-2.0 | Astyanax mexicanus |
| 181 | Microbat | Myoluc2.0 | Myotis lucifugus |
| 182 | Mongolian gerbil | MunDraft-v1.0 | Meriones unguiculatus |
| 183 | Mouse Lemur | Mmur_3.0 | Microcebus murinus |
| 184 | Olive baboon | #N/A | Papio anubis |
| 185 | Pig-tailed macaque | Panu_3.0 | Macaca nemestrina |
| 186 | Polar bear | Mnem_1.0 | Ursus maritimus |
| 187 | Prairie vole | UrsMar_1.0 | Microtus ochrogaster |
| 188 | Rabbit | MicOch1.0 | Oryctolagus cuniculus |
| 189 | Rainbow trout | OryCun2.0 | Oncorhynchus mykiss |
| 190 | Rat | Omyk_1.0 | Rattus norvegicus |
| 191 | Sailfin molly | Rnor_6.0 | Poecilia latipinna |
| 192 | Sheepshead minnow | P_latipinna-1.0 | Cyprinodon variegatus |
| 193 | Shortfin molly | C_variegatus-1.0 | Poecilia mexicana |
| 194 | Sooty mangabey | P_mexicana-1.0 | Cercocebus atys |
| 195 | Squirrel | Caty_1.0 | Ictidomys tridecemlineatus |
| 196 | Swamp eel | SpeTri2.0 | Monopterus albus |
| 197 | Swan goose | M_albus_1.0 | Anser cygnoides |
| 198 | Three-toed box turtle | GooseV1.0 | Terrapene carolina triunguis |
| 199 | Tiger | T_m_triunguis-2.0 | Panthera tigris altaica |
| 200 | Tongue sole | PanTig1.0 | Cynoglossus semilaevis |
| 201 | Wallaby | Cse_v1.0 | Notamacropus eugenii |
| 202 | West African mud turtle | Meug_1.0 | Pelusios castaneus |
| 203 | Wild yak | Pelusios_castaneus-1.0 | Bos mutus |
| 204 | Abingdon island giant tortoise | BosGru_v2.0 | Chelonoidis abingdonii |
| 205 | African ostrich | ASM359739v1 | Struthio camelus australis |
| 206 | Agassiz's desert tortoise | ASM69896v1 | Gopherus agassizii |
| 207 | Algerian mouse | ASM289641v1 | Mus spretus |
| 208 | Alpaca | SPRET_EiJ_v1 | Vicugna pacos |
| 209 | American black bear | vicPac1 | Ursus americanus |
| 210 | Arabian camel | ASM334442v1 | Camelus dromedarius |
| 211 | Arctic ground squirrel | CamDro2 | Urocitellus parryii |
| 212 | Argentine black and white tegu | ASM342692v1 | Salvator merianae |
| 213 | Asiatic black bear | HLtupMer3 | Ursus thibetanus thibetanus |
| 214 | Atlantic salmon | ASM966005v1 | Salmo salar |
| 215 | Australian saltwater crocodile | ICSASG_v2 | Crocodylus porosus |
| 216 | Ballan wrasse | CroPor_comp1 | Labrus bergylta |
| 217 | Barramundi perch | BallGen_V1 | Lates calcarifer |
| 218 | Beluga whale | ASB_HGAPassembly_v1 | Delphinapterus leucas |
| 219 | Bengalese finch | ASM228892v3 | Lonchura striata domestica |
| 220 | Black snub-nosed monkey | LonStrDom1 | Rhinopithecus bieti |
| 221 | Blue tilapia | ASM169854v1 | Oreochromis aureus |
| 222 | Blue tit | ASM587006v1 | Cyanistes caeruleus |
| 223 | Burrowing owl | cyaCae2 | Athene cunicularia |
| 224 | Bushbaby | athCun1 | Otolemur garnettii |
| 225 | C.intestinalis | OtoGar3 | Ciona intestinalis |
| 226 | C. elegans | KH | Caenorhabditis elegans |
| 227 | Chacoan peccary | WBcel235 | Catagonus wagneri |
| 228 | Chicken | CatWag_v2_BIUU_UCD | Gallus gallus |
| 229 | Chilean tinamou | GRCg6a | Nothoprocta perdicaria |
| 230 | Chinese medaka | notPer1 | Oryzias sinensis |
| 231 | Cod | ASM858656v1 | Gadus morhua |
| 232 | Coelacanth | gadMor1 | Latimeria chalumnae |
| 233 | Common canary | LatCha1 | Serinus canaria |
| 234 | Common carp | SCA1 | Cyprinus carpio |
| 235 | Common wombat | German_Mirror_carp_1.0 | Vombatus ursinus |
| 236 | Dark-eyed junco | bare-nosed_wombat_genome_assembly | Junco hyemalis |
| 237 | Daurian ground squirrel | ASM382977v1 | Spermophilus dauricus |
| 238 | Dingo | ASM240643v1 | Canis lupus dingo |
| 239 | Dolphin | ASM325472v1 | Tursiops truncatus |
| 240 | Donkey | - | Equus asinus asinus |
| 241 | Eastern brown snake | ASM303372v1 | Pseudonaja textilis |
| 242 | Electric eel | EBS10Xv2-PRI | Electrophorus electricus |
| 243 | Emu | Ee_SOAP_WITH_SSPACE | Dromaius novaehollandiae |
| 244 | Goat | droNov1 | Capra hircus |
| 245 | Golden snub-nosed monkey | ARS1 | Rhinopithecus roxellana |
| 246 | Golden-collared manakin | Rrox_v1 | Manacus vitellinus |
| 247 | Goldfish | ASM171598v2 | Carassius auratus |
| 248 | Gouldian finch | ASM336829v1 | Erythrura gouldiae |
| 249 | Great spotted kiwi | GouldianFinch | Apteryx haastii |
| 250 | Huchen | aptHaa1 | Hucho hucho |
| 251 | Hyrax | ASM331708v1 | Procavia capensis |
| 252 | Japanese medaka HdrR | proCap1 | Oryzias latipes |
| 253 | Komodo dragon | ASM223471v1 | Varanus komodoensis |
| 254 | Lesser hedgehog tenrec | ASM479886v1 | Echinops telfairi |
| 255 | Little spotted kiwi | TENREC | Apteryx owenii |
| 256 | Macaque | aptOwe1 | Macaca mulatta |
| 257 | Mainland tiger snake | Mmul_10 | Notechis scutatus |
| 258 | Mallard | TS10Xv2-PRI | Anas platyrhynchos |
| 259 | Mangrove rivulus | ASM874695v1 | Kryptolebias marmoratus |
| 260 | Marmoset | ASM164957v1 | Callithrix jacchus |
| 261 | Meerkat | ASM275486v1 | Suricata suricatta |
| 262 | Megabat | meerkat_22Aug2017_6uvM2_HiC | Pteropus vampyrus |
| 263 | Midas cichlid | pteVam1 | Amphilophus citrinellus |
| 264 | Nile tilapia | Midas_v5 | Oreochromis niloticus |
| 265 | Northern pike | O_niloticus_UMD_NMBU | Esox lucius |
| 266 | Ocean sunfish | Eluc_v4 | Mola mola |
| 267 | Okarito brown kiwi | ASM169857v1 | Apteryx rowi |
| 268 | Opossum | aptRow1 | Monodelphis domestica |
| 269 | Orange clownfish | ASM229v1 | Amphiprion percula |
| 270 | Orangutan | Nemo_v1 | Pongo abelii |
| 271 | Panda | PPYG2 | Ailuropoda melanoleuca |
| 272 | Pink-footed goose | ailMel1 | Anser brachyrhynchus |
| 273 | Ring-necked pheasant | ASM259213v1 | Phasianus colchicus |
| 274 | Round goby | ASM414374v1 | Neogobius melanostomus |
| 275 | Ruff | RGoby_Basel_V2 | Calidris pugnax |
| 276 | S. cerevisiae | ASM143184v1 | Saccharomyces cerevisiae |
| 277 | Siberian musk deer | R64-1-1 | Moschus moschiferus |
| 278 | Silver-eye | MosMos_v2_BIUU_UCD | Zosterops lateralis melanops |
| 279 | Sloth | ASM128173v1 | Choloepus hoffmanni |
| 280 | Small tree finch | choHof1 | Camarhynchus parvulus |
| 281 | Sperm whale | STF_HiC | Physeter catodon |
| 282 | Spiny chromis | ASM283717v2 | Acanthochromis polyacanthus |
| 283 | Spoon-billed sandpiper | ASM210954v1 | Calidris pygmaea |
| 284 | Spotted gar | ASM369795v1 | Lepisosteus oculatus |
| 285 | Steppe mouse | LepOcu1 | Mus spicilegus |
| 286 | Tiger tail seahorse | MUSP714 | Hippocampus comes |
| 287 | Tuatara | H_comes_QL1_v1 | Sphenodon punctatus |
| 288 | Turbot | ASM311381v1 | Scophthalmus maximus |
| 289 | Ugandan red Colobus | ASM318616v1 | Piliocolobus tephrosceles |
| 290 | Western mosquitofish | ASM277652v2 | Gambusia affinis |
| 291 | Yellow-billed parrot | ASM309773v1 | Amazona collaria |
| 292 | Yellowtail amberjack | ASM394721v1 | Seriola lalandi dorsalis |
| 293 | Zebra mbuna | Sedor1 | Maylandia zebra |
| 294 | Zebrafish | M_zebra_UMD2a | Danio rerio |
