# Supplementary material for: Identification of upstream transcription factor binding sites in orthologous genes using mixed Student’s t-test statistics
Source: PLoS Comput Biol. 2022 Jun 7;18(6):e1009773. doi: 10.1371/journal.pcbi.1009773 (PMC9205514; doi:10.1371/journal.pcbi.1009773)
Supplement: S2 Table — (DOCX) [file pcbi.1009773.s003.docx]

S2_Table. Detailed information of automated annotated ChIP-Seq datasets obtained from ReMap-2020 database

Table S2. Information of selected ReMap-2020 datasets

| # Index | Motif:sample | Number of TFBS | Transcription factor | Sample set | Information |
| --- | --- | --- | --- | --- | --- |
| 1 | XBP1:set_537 | 21507 | XBP1 | set_537 | XBP1:MDA-MB-231 |
| 2 | RELA:set_892 | 21441 | RELA | set_892 | RELA:monocyte |
| 3 | RUNX2:set_318 | 19778 | RUNX2 | set_318 | RUNX2:IMSC3 |
| 4 | TEAD3:set_423 | 18947 | TEAD3 | set_423 | TEAD3:Hep-G2 |
| 5 | HIF1A:set_290 | 17813 | HIF1A | set_290 | HIF1A:T-47D |
| 6 | TP63:set_690 | 16942 | TP63 | set_690 | TP63:keratinocyte |
| 7 | PBX2:set_214 | 16924 | PBX2 | set_214 | PBX2:K-562 |
| 8 | ESR1:set_378 | 16895 | ESR1 | set_378 | ESR1:MCF-7 |
| 9 | GLIS1:set_94 | 16394 | GLIS1 | set_94 | GLIS1:HEK293 |
| 10 | MYCN:set_831 | 16336 | MYCN | set_831 | MYCN:NGP |
| 11 | GATA2:set_504 | 16055 | GATA2 | set_504 | GATA2:LNCaP |
| 12 | MYC:set_1059 | 15611 | MYC | set_1059 | MYC:LCL |
| 13 | NFIL3:set_484 | 15004 | NFIL3 | set_484 | NFIL3:Hep-G2 |
| 14 | SOX13:set_366 | 14983 | SOX13 | set_366 | SOX13:Hep-G2 |
| 15 | POU2F2:set_1048 | 14957 | POU2F2 | set_1048 | POU2F2:HBL1 |
| 16 | ETV5:set_442 | 14529 | ETV5 | set_442 | ETV5:Hep-G2 |
| 17 | RXRB:set_355 | 13939 | RXRB | set_355 | RXRB:Hep-G2 |
| 18 | RUNX3:set_598 | 13920 | RUNX3 | set_598 | RUNX3:GM12878 |
| 19 | MIXL1:set_455 | 13768 | MIXL1 | set_455 | MIXL1:Hep-G2 |
| 20 | CEBPG:set_428 | 13724 | CEBPG | set_428 | CEBPG:Hep-G2 |
| 21 | HOXB13:set_195 | 13572 | HOXB13 | set_195 | HOXB13:VCaP |
| 22 | SNAI2:set_698 | 13315 | SNAI2 | set_698 | SNAI2:keratinocyte |
| 23 | NFIA:set_398 | 13176 | NFIA | set_398 | NFIA:Hep-G2 |
| 24 | MEF2C:set_383 | 12928 | MEF2C | set_383 | MEF2C:MOLM-13 |
| 25 | EGR2:set_710 | 12656 | EGR2 | set_710 | EGR2:HEK293 |
| 26 | PRDM1:set_86 | 12620 | PRDM1 | set_86 | PRDM1:HEK293 |
| 27 | TFE3:set_1276 | 12434 | TFE3 | set_1276 | TFE3:Hep-G2 |
| 28 | SCRT2:set_1289 | 12290 | SCRT2 | set_1289 | SCRT2:HEK293 |
| 29 | RARA:set_847 | 12146 | RARA | set_847 | RARA:Hep-G2 |
| 30 | ZNF384:set_4038 | 12059 | ZNF384 | set_4038 | ZNF384:HEK293 |
| 31 | NR2F1:set_659 | 11671 | NR2F1 | set_659 | NR2F1:MCF-7 |
| 32 | MYC:set_657 | 11596 | MYC | set_657 | MYC:MCF-7 |
| 33 | PLAG1:set_749 | 11454 | PLAG1 | set_749 | PLAG1:K-562 |
| 34 | SCRT1:set_44 | 11433 | SCRT1 | set_44 | SCRT1:HEK293 |
| 35 | FOXP2:set_535 | 11428 | FOXP2 | set_535 | FOXP2:PFSK1 |
| 36 | ELF3:set_388 | 11392 | ELF3 | set_388 | ELF3:Hep-G2 |
| 37 | PPARG:set_1247 | 11353 | PPARG | set_1247 | PPARG:Hep-G2 |
| 38 | PAX5:set_299 | 10929 | PAX5 | set_299 | PAX5:GM12878 |
| 39 | KLF9:set_391 | 10917 | KLF9 | set_391 | KLF9:GBM1A |
| 40 | OTX2:set_551 | 10889 | OTX2 | set_551 | OTX2:D341-Med |
| 41 | TEAD1:set_369 | 10868 | TEAD1 | set_369 | TEAD1:Hep-G2 |
| 42 | HSF1:set_653 | 10850 | HSF1 | set_653 | HSF1:451Lu |
| 43 | TP53:set_833 | 10828 | TP53 | set_833 | TP53:SW480 |
| 44 | CTCF:set_643 | 10791 | CTCF | set_643 | CTCF:HSPC |
| 45 | POU2F2:set_1423 | 10714 | POU2F2 | set_1423 | POU2F2:GM12878 |
| 46 | MLX:set_2116 | 10711 | MLX | set_2116 | MLX:Hep-G2 |
| 47 | NFKB1:set_412 | 10711 | NFKB1 | set_412 | NFKB1:L1236 |
| 48 | EBF1:set_558 | 10607 | EBF1 | set_558 | EBF1:GM12878 |
| 49 | ZBTB7A:set_347 | 10555 | ZBTB7A | set_347 | ZBTB7A:K-562 |
| 50 | GLIS2:set_677 | 10383 | GLIS2 | set_677 | GLIS2:HEK293 |
| 51 | THAP1:set_399 | 10369 | THAP1 | set_399 | THAP1:K-562 |
| 52 | FOXA1:set_517 | 10129 | FOXA1 | set_517 | FOXA1:VCaP |
| 53 | TFAP2C:set_661 | 10022 | TFAP2C | set_661 | TFAP2C:UCLA1-hESCs |
| 54 | ZNF263:set_303 | 10014 | ZNF263 | set_303 | ZNF263:HEK293 |
| 55 | NFE2:set_609 | 9863 | NFE2 | set_609 | NFE2:ProEs |
| 56 | CEBPA:set_2219 | 9828 | CEBPA | set_2219 | CEBPA:Hep-G2 |
| 57 | OTX2:set_1812 | 9792 | OTX2 | set_1812 | OTX2:D283-Med,D341-Med |
| 58 | RELB:set_0 | 9716 | RELB | set_0 | RELB:GM12878 |
| 59 | SP2:set_472 | 9580 | SP2 | set_472 | SP2:HEK293 |
| 60 | RBPJ:set_842 | 9550 | RBPJ | set_842 | RBPJ:Hep-G2 |
| 61 | TEAD4:set_596 | 9474 | TEAD4 | set_596 | TEAD4:K-562 |
| 62 | NR3C1:set_729 | 9373 | NR3C1 | set_729 | NR3C1:A-549 |
| 63 | TCF3:set_779 | 9245 | TCF3 | set_779 | TCF3:Kasumi-1 |
| 64 | E2F6:set_552 | 9205 | E2F6 | set_552 | E2F6:K-562 |
| 65 | STAT3:set_637 | 9171 | STAT3 | set_637 | STAT3:WA01 |
| 66 | STAT1:set_965 | 9124 | STAT1 | set_965 | STAT1:CD14 |
| 67 | CUX1:set_1260 | 9075 | CUX1 | set_1260 | CUX1:Hep-G2 |
| 68 | ETS1:set_691 | 8855 | ETS1 | set_691 | ETS1:HUVEC-C |
| 69 | IRF2:set_508 | 8817 | IRF2 | set_508 | IRF2:K-562 |
| 70 | MEIS2:set_1061 | 8806 | MEIS2 | set_1061 | MEIS2:K-562 |
| 71 | NEUROG2:set_1034 | 8743 | NEUROG2 | set_1034 | NEUROG2:MRC-5 |
| 72 | NR2F2:set_282 | 8621 | NR2F2 | set_282 | NR2F2:K-562 |
| 73 | GATA6:set_1192 | 8618 | GATA6 | set_1192 | GATA6:PATU8988 |
| 74 | PITX3:set_1023 | 8598 | PITX3 | set_1023 | PITX3:SH-SY5Y |
| 75 | NEUROD1:set_1464 | 8583 | NEUROD1 | set_1464 | NEUROD1:D283-Med |
| 76 | POU4F2:set_2343 | 8582 | POU4F2 | set_2343 | POU4F2:HNPC |
| 77 | PDX1:set_1847 | 8546 | PDX1 | set_1847 | PDX1:islet |
| 78 | ZBTB33:set_116 | 8535 | ZBTB33 | set_116 | ZBTB33:K-562 |
| 79 | CEBPD:set_1197 | 8339 | CEBPD | set_1197 | CEBPD:Hep-G2 |
| 80 | ZEB1:set_556 | 8314 | ZEB1 | set_556 | ZEB1:GM12878 |
| 81 | ETV4:set_1619 | 8299 | ETV4 | set_1619 | ETV4:Hep-G2 |
| 82 | TFAP4:set_622 | 8215 | TFAP4 | set_622 | TFAP4:Hep-G2 |
| 83 | ZNF263:set_778 | 8184 | ZNF263 | set_778 | ZNF263:K-562 |
| 84 | MITF:set_1943 | 8174 | MITF | set_1943 | MITF:melanocyte |
| 85 | ZNF24:set_82 | 8077 | ZNF24 | set_82 | ZNF24:K-562 |
| 86 | TCF3:set_354 | 8028 | TCF3 | set_354 | TCF3:GM12878 |
| 87 | JUN:set_201 | 8012 | JUN | set_201 | JUN:HAEC |
| 88 | SP3:set_66 | 7937 | SP3 | set_66 | SP3:HEK293 |
| 89 | FOXO3:set_2112 | 7934 | FOXO3 | set_2112 | FOXO3:Hep-G2 |
| 90 | MAFF:set_1268 | 7859 | MAFF | set_1268 | MAFF:HeLa-S3 |
| 91 | VDR:set_178 | 7707 | VDR | set_178 | VDR:THP-1 |
| 92 | STAT1:set_1326 | 7696 | STAT1 | set_1326 | STAT1:SET-2 |
| 93 | SOX9:set_345 | 7683 | SOX9 | set_345 | SOX9:HT29 |
| 94 | SRF:set_859 | 7638 | SRF | set_859 | SRF:A-673-clone-Asp114 |
| 95 | STAT3:set_248 | 7621 | STAT3 | set_248 | STAT3:HepaRG |
| 96 | TFAP2A:set_1930 | 7609 | TFAP2A | set_1930 | TFAP2A:MCF-7 |
| 97 | IRF4:set_270 | 7571 | IRF4 | set_270 | IRF4:MM1-S |
| 98 | ERG:set_574 | 7555 | ERG | set_574 | ERG:VCaP |
| 99 | KLF16:set_721 | 7539 | KLF16 | set_721 | KLF16:HEK293 |
| 100 | E2F4:set_587 | 7525 | E2F4 | set_587 | E2F4:K-562 |
| 101 | SP4:set_790 | 7464 | SP4 | set_790 | SP4:HEK293 |
| 102 | PBX3:set_2813 | 7397 | PBX3 | set_2813 | PBX3:GM12878 |
| 103 | JUND:set_597 | 7339 | JUND | set_597 | JUND:K-562 |
| 104 | TGIF2:set_2559 | 7285 | TGIF2 | set_2559 | TGIF2:Hep-G2 |
| 105 | EHF:set_3451 | 7106 | EHF | set_3451 | EHF:RWPE-1 |
| 106 | NEUROD1:set_709 | 7050 | NEUROD1 | set_709 | NEUROD1:D341-Med |
| 107 | RFX3:set_2087 | 7039 | RFX3 | set_2087 | RFX3:Hep-G2 |
| 108 | FOXP1:set_1317 | 7024 | FOXP1 | set_1317 | FOXP1:VCaP |
| 109 | ZSCAN4:set_26 | 7022 | ZSCAN4 | set_26 | ZSCAN4:HEK293 |
| 110 | JUNB:set_410 | 6969 | JUNB | set_410 | JUNB:K-562 |
| 111 | MAX:set_577 | 6969 | MAX | set_577 | MAX:K-562 |
| 112 | SP1:set_625 | 6923 | SP1 | set_625 | SP1:liver |
| 113 | ELF1:set_1528 | 6911 | ELF1 | set_1528 | ELF1:GM12878 |
| 114 | ESR2:set_1994 | 6884 | ESR2 | set_1994 | ESR2:MDA-MB-231 |
| 115 | T:set_1966 | 6868 | T | set_1966 | T:H9 |
| 116 | TBX21:set_1476 | 6842 | TBX21 | set_1476 | TBX21:TH1 |
| 117 | CTCFL:set_154 | 6775 | CTCFL | set_154 | CTCFL:K-562 |
| 118 | ATF4:set_2742 | 6755 | ATF4 | set_2742 | ATF4:K-562 |
| 119 | FOXP1:set_111 | 6716 | FOXP1 | set_111 | FOXP1:H9 |
| 120 | SMAD3:set_805 | 6680 | SMAD3 | set_805 | SMAD3:MDA-MB-231 |
| 121 | EGR1:set_117 | 6594 | EGR1 | set_117 | EGR1:K-562 |
| 122 | CEBPA:set_828 | 6528 | CEBPA | set_828 | CEBPA:Kasumi-1 |
| 123 | HNF4G:set_1244 | 6446 | HNF4G | set_1244 | HNF4G:Hep-G2 |
| 124 | FOSL2:set_1459 | 6432 | FOSL2 | set_1459 | FOSL2:SK-MEL-147 |
| 125 | TCF7L2:set_982 | 6397 | TCF7L2 | set_982 | TCF7L2:HCT-116 |
| 126 | KLF14:set_1169 | 6351 | KLF14 | set_1169 | KLF14:HEK293 |
| 127 | PDX1:set_244 | 6343 | PDX1 | set_244 | PDX1:hiPSC |
| 128 | E2F6:set_548 | 6312 | E2F6 | set_548 | E2F6:WA01 |
| 129 | TWIST1:set_2873 | 6304 | TWIST1 | set_2873 | TWIST1:SK-N-BE2-C,BE2C |
| 130 | TEAD4:set_1542 | 6294 | TEAD4 | set_1542 | TEAD4:WA01 |
| 131 | POU5F1:set_3166 | 6279 | POU5F1 | set_3166 | POU5F1:hESC |
| 132 | NFATC3:set_2054 | 6274 | NFATC3 | set_2054 | NFATC3:GM12878 |
| 133 | YY1:set_1024 | 6268 | YY1 | set_1024 | YY1:HEK293 |
| 134 | FOXK2:set_2075 | 6246 | FOXK2 | set_2075 | FOXK2:K-562 |
| 135 | MZF1:set_1143 | 6164 | MZF1 | set_1143 | MZF1:HEK293 |
| 136 | ESR1:set_553 | 6133 | ESR1 | set_553 | ESR1:breast |
| 137 | POU5F1:set_607 | 6094 | POU5F1 | set_607 | POU5F1:BGO3 |
| 138 | NFATC3:set_663 | 6057 | NFATC3 | set_663 | NFATC3:Hep-G2 |
| 139 | DUX4:set_5891 | 6052 | DUX4 | set_5891 | DUX4:22Rv1 |
| 140 | GMEB2:set_2604 | 6032 | GMEB2 | set_2604 | GMEB2:Hep-G2 |
| 141 | BHLHE40:set_2347 | 6006 | BHLHE40 | set_2347 | BHLHE40:GM12878 |
| 142 | NR4A1:set_1256 | 5983 | NR4A1 | set_1256 | NR4A1:Kasumi-1 |
| 143 | REST:set_714 | 5978 | REST | set_714 | REST:K-562 |
| 144 | PBX3:set_803 | 5976 | PBX3 | set_803 | PBX3:SK-N-SH |
| 145 | SREBF2:set_1032 | 5960 | SREBF2 | set_1032 | SREBF2:HeLa-S3 |
| 146 | MGA:set_1686 | 5958 | MGA | set_1686 | MGA:K-562 |
| 147 | IRF1:set_489 | 5956 | IRF1 | set_489 | IRF1:K-562 |
| 148 | NEUROD1:set_1729 | 5924 | NEUROD1 | set_1729 | NEUROD1:K-562 |
| 149 | SP4:set_1742 | 5878 | SP4 | set_1742 | SP4:WA01 |
| 150 | SOX4:set_1177 | 5868 | SOX4 | set_1177 | SOX4:MRC-5 |
| 151 | KLF5:set_1132 | 5837 | KLF5 | set_1132 | KLF5:HEK293 |
| 152 | NEUROD1:set_940 | 5800 | NEUROD1 | set_940 | NEUROD1:D283-Med,D341-Med |
| 153 | ZNF384:set_3953 | 5799 | ZNF384 | set_3953 | ZNF384:Hep-G2 |
| 154 | USF2:set_1863 | 5790 | USF2 | set_1863 | USF2:IMR-90 |
| 155 | FOXP1:set_1451 | 5782 | FOXP1 | set_1451 | FOXP1:Hep-G2 |
| 156 | ELF3:set_1899 | 5773 | ELF3 | set_1899 | ELF3:Hep-G2,PDAC |
| 157 | CREB1:set_226 | 5768 | CREB1 | set_226 | CREB1:Hep-G2 |
| 158 | HMBOX1:set_2892 | 5765 | HMBOX1 | set_2892 | HMBOX1:K-562 |
| 159 | ZBED1:set_529 | 5753 | ZBED1 | set_529 | ZBED1:K-562 |
| 160 | REST:set_4013 | 5744 | REST | set_4013 | REST:SK-N-SH |
| 161 | ATF7:set_3353 | 5732 | ATF7 | set_3353 | ATF7:GM12878 |
| 162 | PKNOX1:set_2057 | 5728 | PKNOX1 | set_2057 | PKNOX1:GM12878 |
| 163 | NFYA:set_1449 | 5708 | NFYA | set_1449 | NFYA:K-562 |
| 164 | SPI1:set_2234 | 5687 | SPI1 | set_2234 | SPI1:macrophage |
| 165 | IRF4:set_2739 | 5672 | IRF4 | set_2739 | IRF4:GM12878 |
| 166 | CDX2:set_2606 | 5638 | CDX2 | set_2606 | CDX2:CACO2 |
| 167 | POU5F1:set_3563 | 5630 | POU5F1 | set_3563 | POU5F1:fetal |
| 168 | CDX2:set_3044 | 5622 | CDX2 | set_3044 | CDX2:LS180 |
| 169 | SP4:set_498 | 5608 | SP4 | set_498 | SP4:WA01,HEK293 |
| 170 | MEF2A:set_1825 | 5589 | MEF2A | set_1825 | MEF2A:GM12878 |
| 171 | ELF3:set_856 | 5585 | ELF3 | set_856 | ELF3:PDAC |
| 172 | TBX21:set_5014 | 5527 | TBX21 | set_5014 | TBX21:GM12878 |
| 173 | EBF1:set_3297 | 5422 | EBF1 | set_3297 | EBF1:MUTUL |
| 174 | ERG:set_1485 | 5402 | ERG | set_1485 | ERG:LNCaP |
| 175 | JUND:set_2147 | 5384 | JUND | set_2147 | JUND:Hep-G2 |
| 176 | NFKB1:set_203 | 5366 | NFKB1 | set_203 | NFKB1:MCF10A-Er-Src |
| 177 | FOSL2:set_530 | 5355 | FOSL2 | set_530 | FOSL2:A-549 |
| 178 | CLOCK:set_2335 | 5353 | CLOCK | set_2335 | CLOCK:MCF-7 |
| 179 | IRF1:set_3023 | 5331 | IRF1 | set_3023 | IRF1:PDAC |
| 180 | PBX1:set_1057 | 5326 | PBX1 | set_1057 | PBX1:RCH-ACV |
| 181 | TCF4:set_1977 | 5269 | TCF4 | set_1977 | TCF4:GEN2-2 |
| 182 | KLF4:set_1369 | 5238 | KLF4 | set_1369 | KLF4:BJ |
| 183 | NR2F2:set_1929 | 5231 | NR2F2 | set_1929 | NR2F2:liver |
| 184 | TP53:set_961 | 5223 | TP53 | set_961 | TP53:IMR-90 |
| 185 | JUN:set_1331 | 5204 | JUN | set_1331 | JUN:786-O |
| 186 | ZNF143:set_570 | 5204 | ZNF143 | set_570 | ZNF143:FLP143HA |
| 187 | CREB3L1:set_2265 | 5201 | CREB3L1 | set_2265 | CREB3L1:K-562 |
| 188 | ZNF143:set_668 | 5173 | ZNF143 | set_668 | ZNF143:MCF-7 |
| 189 | TBX2:set_759 | 5160 | TBX2 | set_759 | TBX2:Kelly |
| 190 | MAX:set_208 | 5124 | MAX | set_208 | MAX:WA01 |
| 191 | MNT:set_59 | 5123 | MNT | set_59 | MNT:K-562 |
| 192 | YY1:set_3922 | 5111 | YY1 | set_3922 | YY1:liver |
| 193 | FOXA1:set_8973 | 5082 | FOXA1 | set_8973 | FOXA1:DU145 |
| 194 | JUNB:set_5977 | 5044 | JUNB | set_5977 | JUNB:MCF10A-Er-Src |
| 195 | GATA3:set_1633 | 5032 | GATA3 | set_1633 | GATA3:T-47D |
| 196 | PROX1:set_781 | 5026 | PROX1 | set_781 | PROX1:SW480 |
| 197 | MYCN:set_711 | 5007 | MYCN | set_711 | MYCN:Kelly |
| 198 | BHLHE40:set_1893 | 4984 | BHLHE40 | set_1893 | BHLHE40:IMR-90 |
| 199 | ATF7:set_1091 | 4970 | ATF7 | set_1091 | ATF7:K-562 |
| 200 | TCF4:set_821 | 4962 | TCF4 | set_821 | TCF4:GEN2-2,CAL-1 |
| 201 | SPI1:set_197 | 4952 | SPI1 | set_197 | SPI1:LCL |
| 202 | KLF4:set_281 | 4907 | KLF4 | set_281 | KLF4:keratinocyte |
| 203 | MAFG:set_2890 | 4895 | MAFG | set_2890 | MAFG:K-562 |
| 204 | NRF1:set_2795 | 4891 | NRF1 | set_2795 | NRF1:HeLa-S3 |
| 205 | NR3C1:set_712 | 4886 | NR3C1 | set_712 | NR3C1:HASM2 |
| 206 | E2F7:set_1265 | 4855 | E2F7 | set_1265 | E2F7:IMR-90 |
| 207 | LEF1:set_80 | 4829 | LEF1 | set_80 | LEF1:K-562 |
| 208 | NFIC:set_136 | 4817 | NFIC | set_136 | NFIC:K-562 |
| 209 | GATA3:set_943 | 4796 | GATA3 | set_943 | GATA3:MCF-7 |
| 210 | USF1:set_2233 | 4793 | USF1 | set_2233 | USF1:SK-N-SH |
| 211 | HLF:set_2776 | 4787 | HLF | set_2776 | HLF:Hep-G2 |
| 212 | RFX5:set_1955 | 4781 | RFX5 | set_1955 | RFX5:HeLa-S3 |
| 213 | ETV6:set_3479 | 4730 | ETV6 | set_3479 | ETV6:GM12878 |
| 214 | PAX5:set_2176 | 4727 | PAX5 | set_2176 | PAX5:GM12878,GM12892 |
| 215 | SREBF1:set_541 | 4722 | SREBF1 | set_541 | SREBF1:K-562 |
| 216 | YY1:set_602 | 4668 | YY1 | set_602 | YY1:GM12878 |
| 217 | SMAD3:set_732 | 4645 | SMAD3 | set_732 | SMAD3:BGO3 |
| 218 | CEBPD:set_814 | 4628 | CEBPD | set_814 | CEBPD:K-562 |
| 219 | ELF4:set_2086 | 4618 | ELF4 | set_2086 | ELF4:K-562 |
| 220 | TFAP2C:set_1107 | 4597 | TFAP2C | set_1107 | TFAP2C:UCLA1-hESCs,A-375,MDA-MB-453,BT-474,MCF-7,SKBR3 |
| 221 | FOXA1:set_567 | 4591 | FOXA1 | set_567 | FOXA1:LNCaP |
| 222 | KLF4:set_1635 | 4570 | KLF4 | set_1635 | KLF4:PDAC |
| 223 | TEAD1:set_1624 | 4558 | TEAD1 | set_1624 | TEAD1:H69 |
| 224 | SP1:set_772 | 4523 | SP1 | set_772 | SP1:HEK293 |
| 225 | ZBTB7B:set_1301 | 4499 | ZBTB7B | set_1301 | ZBTB7B:MCF-7 |
| 226 | MYB:set_3702 | 4475 | MYB | set_3702 | MYB:THP-1 |
| 227 | MEF2B:set_2379 | 4472 | MEF2B | set_2379 | MEF2B:GM12878 |
| 228 | ZNF384:set_515 | 4472 | ZNF384 | set_515 | ZNF384:K-562 |
| 229 | E2F4:set_509 | 4452 | E2F4 | set_509 | E2F4:MCF-10A,K-562 |
| 230 | TBX5:set_3927 | 4449 | TBX5 | set_3927 | TBX5:cardiomyocyte |
| 231 | E2F1:set_3365 | 4446 | E2F1 | set_3365 | E2F1:MDA-MB-231 |
| 232 | RELA:set_700 | 4437 | RELA | set_700 | RELA:786-O |
| 233 | FLI1:set_630 | 4410 | FLI1 | set_630 | FLI1:SKNO1 |
| 234 | RARA:set_580 | 4387 | RARA | set_580 | RARA:TSU-1621MT |
| 235 | E2F8:set_5171 | 4348 | E2F8 | set_5171 | E2F8:GM12878 |
| 236 | GATA2:set_871 | 4338 | GATA2 | set_871 | GATA2:SKH1 |
| 237 | NFE2:set_2919 | 4330 | NFE2 | set_2919 | NFE2:K-562 |
| 238 | E2F6:set_1511 | 4303 | E2F6 | set_1511 | E2F6:K-562,A-549,WA01 |
| 239 | E2F8:set_34 | 4295 | E2F8 | set_34 | E2F8:K-562 |
| 240 | NFIC:set_1886 | 4278 | NFIC | set_1886 | NFIC:Ishikawa |
| 241 | CEBPB:set_3531 | 4268 | CEBPB | set_3531 | CEBPB:LS180 |
| 242 | MXI1:set_1493 | 4246 | MXI1 | set_1493 | MXI1:IMR-90 |
| 243 | MXI1:set_3187 | 4237 | MXI1 | set_3187 | MXI1:SK-N-SH |
| 244 | FOSL1:set_1470 | 4234 | FOSL1 | set_1470 | FOSL1:K-562 |
| 245 | POU2F2:set_2132 | 4227 | POU2F2 | set_2132 | POU2F2:HBL1,GM12878 |
| 246 | HOXB13:set_3882 | 4211 | HOXB13 | set_3882 | HOXB13:VCaP,LNCaP |
| 247 | NR2F2:set_2574 | 4203 | NR2F2 | set_2574 | NR2F2:MCF-7 |
| 248 | RARA:set_439 | 4188 | RARA | set_439 | RARA:Hep-G2,TSU-1621MT |
| 249 | FLI1:set_2467 | 4174 | FLI1 | set_2467 | FLI1:A-673-clone-Asp114 |
| 250 | EGR1:set_1280 | 4140 | EGR1 | set_1280 | EGR1:A-375 |
| 251 | ERG:set_4603 | 4131 | ERG | set_4603 | ERG:LNCaP,VCaP |
| 252 | E2F6:set_2136 | 4100 | E2F6 | set_2136 | E2F6:K-562,WA01 |
| 253 | CREB1:set_1862 | 4099 | CREB1 | set_1862 | CREB1:WA01 |
| 254 | MNT:set_2061 | 4089 | MNT | set_2061 | MNT:MCF-7,Hep-G2,K-562 |
| 255 | CTCFL:set_971 | 4089 | CTCFL | set_971 | CTCFL:K-562,OVCAR-8 |
| 256 | YY1:set_979 | 4053 | YY1 | set_979 | YY1:K-562 |
| 257 | PPARG:set_296 | 4040 | PPARG | set_296 | PPARG:HUVEC-C |
| 258 | SPIB:set_3700 | 4040 | SPIB | set_3700 | SPIB:OCI-Ly3 |
| 259 | NR2F1:set_4610 | 4022 | NR2F1 | set_4610 | NR2F1:K-562 |
| 260 | MEF2B:set_4775 | 4009 | MEF2B | set_4775 | MEF2B:tonsil |
| 261 | ZNF263:set_832 | 3985 | ZNF263 | set_832 | ZNF263:HEK293,K-562 |
| 262 | CLOCK:set_2349 | 3980 | CLOCK | set_2349 | CLOCK:BA40-neocortex |
| 263 | GATA3:set_415 | 3942 | GATA3 | set_415 | GATA3:Jurkat |
| 264 | OTX2:set_6093 | 3941 | OTX2 | set_6093 | OTX2:D283-Med |
| 265 | TEAD4:set_2312 | 3936 | TEAD4 | set_2312 | TEAD4:SK-MEL-147 |
| 266 | HOXB13:set_4011 | 3933 | HOXB13 | set_4011 | HOXB13:LNCaP |
| 267 | CTCFL:set_2613 | 3929 | CTCFL | set_2613 | CTCFL:OVCAR-8 |
| 268 | REST:set_948 | 3917 | REST | set_948 | REST:A-549 |
| 269 | KLF13:set_1355 | 3899 | KLF13 | set_1355 | KLF13:K-562 |
| 270 | HSF1:set_618 | 3896 | HSF1 | set_618 | HSF1:MO91 |
| 271 | TEAD1:set_1425 | 3891 | TEAD1 | set_1425 | TEAD1:MCF-7 |
| 272 | KLF9:set_1066 | 3891 | KLF9 | set_1066 | KLF9:GBM1A,HEK293 |
| 273 | SOX4:set_171 | 3889 | SOX4 | set_171 | SOX4:MDA-MB-231 |
| 274 | CTCF:set_2288 | 3881 | CTCF | set_2288 | CTCF:HEK293 |
| 275 | PDX1:set_1068 | 3864 | PDX1 | set_1068 | PDX1:islet,hiPSC |
| 276 | TBX21:set_2137 | 3858 | TBX21 | set_2137 | TBX21:TH1,CD4 |
| 277 | IRF4:set_409 | 3845 | IRF4 | set_409 | IRF4:GM12878,MM1-S |
| 278 | CEBPD:set_429 | 3839 | CEBPD | set_429 | CEBPD:Hep-G2,K-562 |
| 279 | TFDP1:set_665 | 3820 | TFDP1 | set_665 | TFDP1:K-562 |
| 280 | NFKB2:set_3035 | 3814 | NFKB2 | set_3035 | NFKB2:L1236 |
| 281 | PAX5:set_7022 | 3806 | PAX5 | set_7022 | PAX5:fetal |
| 282 | ZNF143:set_3863 | 3799 | ZNF143 | set_3863 | ZNF143:FLP76 |
| 283 | ELK4:set_2083 | 3780 | ELK4 | set_2083 | ELK4:HeLa-S3 |
| 284 | JUND:set_3244 | 3766 | JUND | set_3244 | JUND:WA01 |
| 285 | SMAD3:set_1846 | 3761 | SMAD3 | set_1846 | SMAD3:NCI-H441 |
| 286 | NFE2:set_2144 | 3748 | NFE2 | set_2144 | NFE2:ProEs,erythroid |
| 287 | BHLHE40:set_2101 | 3727 | BHLHE40 | set_2101 | BHLHE40:Hep-G2,GM12878,IMR-90,K-562 |
| 288 | NFYB:set_268 | 3721 | NFYB | set_268 | NFYB:K-562 |
| 289 | YY2:set_727 | 3701 | YY2 | set_727 | YY2:HEK293 |
| 290 | ZNF282:set_1090 | 3658 | ZNF282 | set_1090 | ZNF282:K-562 |
| 291 | NR2C2:set_2317 | 3647 | NR2C2 | set_2317 | NR2C2:K-562 |
| 292 | MYC:set_1498 | 3645 | MYC | set_1498 | MYC:P493-6 |
| 293 | TEAD1:set_4439 | 3634 | TEAD1 | set_4439 | TEAD1:T-47D |
| 294 | ZNF24:set_1856 | 3605 | ZNF24 | set_1856 | ZNF24:HEK293 |
| 295 | JUN:set_1904 | 3604 | JUN | set_1904 | JUN:MDA-MB-231 |
| 296 | RBPJ:set_2457 | 3587 | RBPJ | set_2457 | RBPJ:GSC8-11 |
| 297 | ERG:set_176 | 3586 | ERG | set_176 | ERG:SKNO1 |
| 298 | ETV1:set_632 | 3582 | ETV1 | set_632 | ETV1:MDA-Pca-2b |
| 299 | JUN:set_245 | 3561 | JUN | set_245 | JUN:MCF10A-Er-Src |
| 300 | MAFF:set_5224 | 3561 | MAFF | set_5224 | MAFF:Hep-G2 |
| 301 | NR2F1:set_77 | 3539 | NR2F1 | set_77 | NR2F1:GM12878 |
| 302 | SP1:set_1921 | 3537 | SP1 | set_1921 | SP1:GM12878 |
| 303 | FOXP1:set_164 | 3527 | FOXP1 | set_164 | FOXP1:LNCaP |
| 304 | TP53:set_377 | 3522 | TP53 | set_377 | TP53:MDA-MB-231,SW480 |
| 305 | MYCN:set_435 | 3522 | MYCN | set_435 | MYCN:BE2C,Kelly,SHEP-21N,NGP,SK-N-BE2-C |
| 306 | GATA6:set_294 | 3519 | GATA6 | set_294 | GATA6:AGS |
| 307 | NFE2:set_2141 | 3518 | NFE2 | set_2141 | NFE2:erythroid |
| 308 | MAX:set_1336 | 3516 | MAX | set_1336 | MAX:MKL-1 |
| 309 | ERG:set_846 | 3506 | ERG | set_846 | ERG:Jurkat |
| 310 | ETV1:set_108 | 3501 | ETV1 | set_108 | ETV1:GIST-T1 |
| 311 | FOS:set_2684 | 3500 | FOS | set_2684 | FOS:Hep-G2 |
| 312 | NR3C1:set_253 | 3491 | NR3C1 | set_253 | NR3C1:HeLa-B2 |
| 313 | SPI1:set_2023 | 3475 | SPI1 | set_2023 | SPI1:monocyte |
| 314 | PRDM1:set_4194 | 3474 | PRDM1 | set_4194 | PRDM1:fetal |
| 315 | GATA3:set_3775 | 3461 | GATA3 | set_3775 | GATA3:breast |
| 316 | ESR1:set_2297 | 3450 | ESR1 | set_2297 | ESR1:Ishikawa |
| 317 | CEBPB:set_887 | 3443 | CEBPB | set_887 | CEBPB:Hep-G2 |
| 318 | CTCFL:set_1124 | 3442 | CTCFL | set_1124 | CTCFL:delta-47,K-562,OVCAR-8 |
| 319 | CEBPB:set_1286 | 3416 | CEBPB | set_1286 | CEBPB:HeLa-S3 |
| 320 | FOS:set_2994 | 3413 | FOS | set_2994 | FOS:MCF10A-Er-Src |
| 321 | ZBTB7A:set_229 | 3409 | ZBTB7A | set_229 | ZBTB7A:Hep-G2 |
| 322 | REST:set_2437 | 3402 | REST | set_2437 | REST:neural |
| 323 | GRHL2:set_1044 | 3395 | GRHL2 | set_1044 | GRHL2:MCF-7-TAMR-1 |
| 324 | SIX2:set_1788 | 3394 | SIX2 | set_1788 | SIX2:HEK,kidney |
| 325 | TWIST1:set_1046 | 3393 | TWIST1 | set_1046 | TWIST1:SK-N-BE2-C,BE2C,SHEP-21N |
| 326 | NEUROD1:set_320 | 3391 | NEUROD1 | set_320 | NEUROD1:D283-Med,K-562,D341-Med |
| 327 | ELF1:set_2380 | 3387 | ELF1 | set_2380 | ELF1:K-562 |
| 328 | KLF9:set_40 | 3371 | KLF9 | set_40 | KLF9:HEK293 |
| 329 | FLI1:set_2750 | 3349 | FLI1 | set_2750 | FLI1:A-673 |
| 330 | TCF3:set_1418 | 3345 | TCF3 | set_1418 | TCF3:RCH-ACV,GM12878 |
| 331 | FOXA1:set_981 | 3311 | FOXA1 | set_981 | FOXA1:MCF-7 |
| 332 | MITF:set_912 | 3304 | MITF | set_912 | MITF:K-562 |
| 333 | KLF16:set_1778 | 3289 | KLF16 | set_1778 | KLF16:K-562 |
| 334 | NR3C1:set_3234 | 3279 | NR3C1 | set_3234 | NR3C1:Ishikawa |
| 335 | CTCF:set_1302 | 3255 | CTCF | set_1302 | CTCF:MCF-7 |
| 336 | ONECUT1:set_1618 | 3255 | ONECUT1 | set_1618 | ONECUT1:liver |
| 337 | MNT:set_2301 | 3255 | MNT | set_2301 | MNT:Hep-G2 |
| 338 | SP2:set_2114 | 3254 | SP2 | set_2114 | SP2:HEK293,HEK293T |
| 339 | POU5F1:set_4792 | 3254 | POU5F1 | set_4792 | POU5F1:BJ |
| 340 | NR4A1:set_2452 | 3249 | NR4A1 | set_2452 | NR4A1:K-562 |
| 341 | JUN:set_1683 | 3246 | JUN | set_1683 | JUN:K-562 |
| 342 | ZBTB7A:set_735 | 3236 | ZBTB7A | set_735 | ZBTB7A:Ishikawa |
| 343 | CREB1:set_1680 | 3235 | CREB1 | set_1680 | CREB1:MCF-7 |
| 344 | USF2:set_680 | 3229 | USF2 | set_680 | USF2:K-562 |
| 345 | KLF5:set_3705 | 3226 | KLF5 | set_3705 | KLF5:KATOIII |
| 346 | ZNF143:set_1644 | 3222 | ZNF143 | set_1644 | ZNF143:GM12878 |
| 347 | PAX5:set_1381 | 3221 | PAX5 | set_1381 | PAX5:GM12892 |
| 348 | FOSL2:set_3131 | 3220 | FOSL2 | set_3131 | FOSL2:Hep-G2 |
| 349 | PKNOX1:set_2367 | 3215 | PKNOX1 | set_2367 | PKNOX1:K-562 |
| 350 | ZNF384:set_2865 | 3191 | ZNF384 | set_2865 | ZNF384:K-562,Hep-G2 |
| 351 | E2F1:set_616 | 3172 | E2F1 | set_616 | E2F1:U-87MG |
| 352 | CEBPB:set_1087 | 3171 | CEBPB | set_1087 | CEBPB:MV4-11 |
| 353 | TFAP2C:set_153 | 3141 | TFAP2C | set_153 | TFAP2C:A-375 |
| 354 | POU5F1:set_152 | 3125 | POU5F1 | set_152 | POU5F1:BJ1-hTERT |
| 355 | E2F4:set_2565 | 3122 | E2F4 | set_2565 | E2F4:MCF-10A |
| 356 | YY1:set_3259 | 3122 | YY1 | set_3259 | YY1:WA01 |
| 357 | CEBPA:set_1417 | 3113 | CEBPA | set_1417 | CEBPA:MV4-11 |
| 358 | JUNB:set_7728 | 3111 | JUNB | set_7728 | JUNB:GM12878 |
| 359 | NFIC:set_3835 | 3109 | NFIC | set_3835 | NFIC:GM12878 |
| 360 | GATA3:set_4049 | 3092 | GATA3 | set_4049 | GATA3:SK-N-SH |
| 361 | FOS:set_5063 | 3088 | FOS | set_5063 | FOS:MCF10A-Er-Src,MCF-10A |
| 362 | NR3C1:set_2303 | 3069 | NR3C1 | set_2303 | NR3C1:breast |
| 363 | TEAD4:set_2384 | 3046 | TEAD4 | set_2384 | TEAD4:Ishikawa |
| 364 | FOXA1:set_3396 | 3044 | FOXA1 | set_3396 | FOXA1:breast |
| 365 | ETS1:set_599 | 3021 | ETS1 | set_599 | ETS1:K-562 |
| 366 | TWIST1:set_1320 | 2996 | TWIST1 | set_1320 | TWIST1:SHEP-21N |
| 367 | JUNB:set_196 | 2990 | JUNB | set_196 | JUNB:HAEC |
| 368 | POU5F1:set_767 | 2988 | POU5F1 | set_767 | POU5F1:WA01 |
| 369 | CDX2:set_1249 | 2983 | CDX2 | set_1249 | CDX2:LS180,CACO2 |
| 370 | BHLHE22:set_1051 | 2972 | BHLHE22 | set_1051 | BHLHE22:CAL-1 |
| 371 | GATA2:set_2010 | 2967 | GATA2 | set_2010 | GATA2:K-562 |
| 372 | GRHL2:set_9776 | 2963 | GRHL2 | set_9776 | GRHL2:MCF-7 |
| 373 | MAFK:set_173 | 2962 | MAFK | set_173 | MAFK:OCI-Ly7 |
| 374 | FOXP1:set_1961 | 2957 | FOXP1 | set_1961 | FOXP1:Hep-G2,H9 |
| 375 | EOMES:set_2877 | 2914 | EOMES | set_2877 | EOMES:hESC |
| 376 | SPDEF:set_2328 | 2907 | SPDEF | set_2328 | SPDEF:A-549 |
| 377 | TBX5:set_626 | 2899 | TBX5 | set_626 | TBX5:G296S |
| 378 | MYCN:set_1585 | 2894 | MYCN | set_1585 | MYCN:SHEP-21N |
| 379 | JUND:set_167 | 2888 | JUND | set_167 | JUND:GP5D |
| 380 | ZBTB33:set_2931 | 2883 | ZBTB33 | set_2931 | ZBTB33:liver |
| 381 | CEBPG:set_2896 | 2878 | CEBPG | set_2896 | CEBPG:K-562,Hep-G2 |
| 382 | STAT1:set_390 | 2876 | STAT1 | set_390 | STAT1:CD14,SET-2 |
| 383 | TCF4:set_1304 | 2843 | TCF4 | set_1304 | TCF4:CAL-1 |
| 384 | CEBPA:set_3084 | 2833 | CEBPA | set_3084 | CEBPA:SKH1 |
| 385 | PBX3:set_1779 | 2827 | PBX3 | set_1779 | PBX3:GM12878,A-549,SK-N-SH |
| 386 | RELA:set_1201 | 2824 | RELA | set_1201 | RELA:SGBS |
| 387 | ESR1:set_2947 | 2819 | ESR1 | set_2947 | ESR1:breast,MCF-7 |
| 388 | TCF3:set_921 | 2819 | TCF3 | set_921 | TCF3:RCH-ACV |
| 389 | STAT3:set_5199 | 2809 | STAT3 | set_5199 | STAT3:MCF-10A |
| 390 | GATA6:set_10476 | 2808 | GATA6 | set_10476 | GATA6:WA01 |
| 391 | CREB1:set_1519 | 2807 | CREB1 | set_1519 | CREB1:LNCaP-abl |
| 392 | PAX5:set_2122 | 2805 | PAX5 | set_2122 | PAX5:GM12878,GM12892,GM12891 |
| 393 | NFATC3:set_2891 | 2798 | NFATC3 | set_2891 | NFATC3:K-562 |
| 394 | KLF13:set_3522 | 2783 | KLF13 | set_3522 | KLF13:HEK293 |
| 395 | MAX:set_4569 | 2779 | MAX | set_4569 | MAX:MDA-MB-468 |
| 396 | E2F8:set_2894 | 2778 | E2F8 | set_2894 | E2F8:K-562,GM12878 |
| 397 | FLI1:set_3836 | 2770 | FLI1 | set_3836 | FLI1:A-673-clone-Asp114,A-673 |
| 398 | REST:set_920 | 2769 | REST | set_920 | REST:PANC-1 |
| 399 | SP1:set_4925 | 2750 | SP1 | set_4925 | SP1:WA01 |
| 400 | ZEB1:set_1752 | 2736 | ZEB1 | set_1752 | ZEB1:HEK293 |
| 401 | CEBPG:set_6640 | 2733 | CEBPG | set_6640 | CEBPG:K-562 |
| 402 | BHLHE40:set_519 | 2722 | BHLHE40 | set_519 | BHLHE40:K-562 |
| 403 | NR2F1:set_1385 | 2715 | NR2F1 | set_1385 | NR2F1:MCF-7,GM12878 |
| 404 | CLOCK:set_1906 | 2712 | CLOCK | set_1906 | CLOCK:U2OS |
| 405 | RELA:set_671 | 2709 | RELA | set_671 | RELA:HEK293 |
| 406 | USF1:set_634 | 2704 | USF1 | set_634 | USF1:WA01 |
| 407 | RELA:set_2886 | 2696 | RELA | set_2886 | RELA:LNCaP |
| 408 | CREB3:set_2710 | 2696 | CREB3 | set_2710 | CREB3:K-562 |
| 409 | FOXA1:set_613 | 2687 | FOXA1 | set_613 | FOXA1:MDA-MB-453 |
| 410 | BHLHE40:set_224 | 2686 | BHLHE40 | set_224 | BHLHE40:Hep-G2 |
| 411 | ZNF24:set_1229 | 2679 | ZNF24 | set_1229 | ZNF24:K-562,HEK293 |
| 412 | REST:set_4211 | 2676 | REST | set_4211 | REST:liver |
| 413 | NRF1:set_1187 | 2670 | NRF1 | set_1187 | NRF1:K-562 |
| 414 | RELA:set_205 | 2668 | RELA | set_205 | RELA:HeLa-B2 |
| 415 | ZNF143:set_2806 | 2666 | ZNF143 | set_2806 | ZNF143:WA01 |
| 416 | MAX:set_566 | 2666 | MAX | set_566 | MAX:U-87MG |
| 417 | NFYA:set_467 | 2663 | NFYA | set_467 | NFYA:HeLa-S3,K-562 |
| 418 | ZEB1:set_788 | 2660 | ZEB1 | set_788 | ZEB1:PDAC |
| 419 | SPI1:set_5184 | 2656 | SPI1 | set_5184 | SPI1:THP-1 |
| 420 | CLOCK:set_4040 | 2639 | CLOCK | set_4040 | CLOCK:BA10-neocortex |
| 421 | STAT3:set_1678 | 2618 | STAT3 | set_1678 | STAT3:HCC70 |
| 422 | CREB1:set_2364 | 2617 | CREB1 | set_2364 | CREB1:A-549 |
| 423 | CEBPB:set_285 | 2614 | CEBPB | set_285 | CEBPB:K-562 |
| 424 | DUX4:set_7614 | 2612 | DUX4 | set_7614 | DUX4:WA01,22Rv1 |
| 425 | GRHL2:set_1537 | 2611 | GRHL2 | set_1537 | GRHL2:MCF-7,MCF-7-TAMR-1,MCF-7-WS8 |
| 426 | SMAD3:set_3057 | 2610 | SMAD3 | set_3057 | SMAD3:HCC1954 |
| 427 | HNF1A:set_3331 | 2584 | HNF1A | set_3331 | HNF1A:Hep-G2 |
| 428 | RFX5:set_1308 | 2582 | RFX5 | set_1308 | RFX5:Hep-G2 |
| 429 | FOXA1:set_3853 | 2578 | FOXA1 | set_3853 | FOXA1:VCaP,LNCaP |
| 430 | FLI1:set_638 | 2570 | FLI1 | set_638 | FLI1:TSU-1621MT |
| 431 | NFIC:set_1941 | 2569 | NFIC | set_1941 | NFIC:SK-N-SH |
| 432 | ELF1:set_3430 | 2553 | ELF1 | set_3430 | ELF1:Hep-G2 |
| 433 | VDR:set_2804 | 2548 | VDR | set_2804 | VDR:LNCaP |
| 434 | HNF1B:set_875 | 2539 | HNF1B | set_875 | HNF1B:PDAC |
| 435 | TBX21:set_6454 | 2537 | TBX21 | set_6454 | TBX21:TH1,GM12878,CD4 |
| 436 | CTCF:set_8567 | 2536 | CTCF | set_8567 | CTCF:LUHMES |
| 437 | TFAP2C:set_2289 | 2534 | TFAP2C | set_2289 | TFAP2C:MDA-MB-453 |
| 438 | MAFK:set_3511 | 2515 | MAFK | set_3511 | MAFK:IMR-90 |
| 439 | GATA3:set_936 | 2511 | GATA3 | set_936 | GATA3:MCF-7,T-47D |
| 440 | RUNX2:set_631 | 2502 | RUNX2 | set_631 | RUNX2:LNCaP-C4-2B |
| 441 | PBX3:set_3199 | 2500 | PBX3 | set_3199 | PBX3:GM12878,SK-N-SH |
| 442 | RELA:set_3421 | 2498 | RELA | set_3421 | RELA:HAEC |
| 443 | MAFF:set_5718 | 2491 | MAFF | set_5718 | MAFF:HeLa-S3,Hep-G2 |
| 444 | ASCL1:set_1165 | 2488 | ASCL1 | set_1165 | ASCL1:NCI-H82 |
| 445 | ZBTB33:set_3224 | 2481 | ZBTB33 | set_3224 | ZBTB33:GM12878 |
| 446 | NFYB:set_437 | 2474 | NFYB | set_437 | NFYB:HeLa-S3,K-562,GM12878 |
| 447 | CEBPB:set_2875 | 2474 | CEBPB | set_2875 | CEBPB:epididymis |
| 448 | HIF1A:set_11034 | 2469 | HIF1A | set_11034 | HIF1A:U2OS |
| 449 | ERF:set_3838 | 2468 | ERF | set_3838 | ERF:HAEC |
| 450 | TCF3:set_3144 | 2451 | TCF3 | set_3144 | TCF3:Kasumi-1,GM12878 |
| 451 | SRF:set_3982 | 2429 | SRF | set_3982 | SRF:Ishikawa |
| 452 | CREB1:set_3556 | 2426 | CREB1 | set_3556 | CREB1:GM12878 |
| 453 | ZEB1:set_994 | 2420 | ZEB1 | set_994 | ZEB1:neuron |
| 454 | TFDP1:set_406 | 2418 | TFDP1 | set_406 | TFDP1:MM1-S |
| 455 | MNT:set_1030 | 2405 | MNT | set_1030 | MNT:MCF-7 |
| 456 | MYC:set_877 | 2405 | MYC | set_877 | MYC:CD34 |
| 457 | ZNF143:set_3526 | 2393 | ZNF143 | set_3526 | ZNF143:K-562 |
| 458 | MYB:set_830 | 2385 | MYB | set_830 | MYB:MOLT-3 |
| 459 | MYBL2:set_4969 | 2381 | MYBL2 | set_4969 | MYBL2:Hep-G2 |
| 460 | KLF13:set_1190 | 2379 | KLF13 | set_1190 | KLF13:K-562,HEK293 |
| 461 | MYC:set_2146 | 2373 | MYC | set_2146 | MYC:K-562 |
| 462 | CTCF:set_3562 | 2354 | CTCF | set_3562 | CTCF:GM12892 |
| 463 | ERG:set_5789 | 2349 | ERG | set_5789 | ERG:TSU-1621MT |
| 464 | ZNF143:set_898 | 2338 | ZNF143 | set_898 | ZNF143:Hep-G2 |
| 465 | FOXA1:set_3525 | 2337 | FOXA1 | set_3525 | FOXA1:Hep-G2 |
| 466 | HNF4G:set_2005 | 2335 | HNF4G | set_2005 | HNF4G:22Rv1 |
| 467 | TP53:set_3809 | 2329 | TP53 | set_3809 | TP53:MDA-MB-231 |
| 468 | PKNOX1:set_1065 | 2320 | PKNOX1 | set_1065 | PKNOX1:HEK293T,MCF-7,GM12878,K-562 |
| 469 | ESR1:set_939 | 2305 | ESR1 | set_939 | ESR1:MSF-7,MCF-7 |
| 470 | TCF4:set_3981 | 2293 | TCF4 | set_3981 | TCF4:LS180 |
| 471 | SRF:set_3086 | 2287 | SRF | set_3086 | SRF:GM12878 |
| 472 | TFAP2C:set_4774 | 2286 | TFAP2C | set_4774 | TFAP2C:BT-474 |
| 473 | VDR:set_1754 | 2276 | VDR | set_1754 | VDR:LX2 |
| 474 | GATA2:set_1467 | 2264 | GATA2 | set_1467 | GATA2:LNCaP,SKH1 |
| 475 | MAFK:set_2802 | 2263 | MAFK | set_2802 | MAFK:Hep-G2 |
| 476 | MEIS1:set_6502 | 2261 | MEIS1 | set_6502 | MEIS1:SEM |
| 477 | MYB:set_919 | 2252 | MYB | set_919 | MYB:Jurkat |
| 478 | SP1:set_904 | 2248 | SP1 | set_904 | SP1:A-549 |
| 479 | NFIA:set_1225 | 2247 | NFIA | set_1225 | NFIA:Hep-G2,K-562 |
| 480 | YY2:set_2094 | 2239 | YY2 | set_2094 | YY2:HEK293,HeLa |
| 481 | MAX:set_820 | 2228 | MAX | set_820 | MAX:NCI-H2171 |
| 482 | TFAP2C:set_1483 | 2227 | TFAP2C | set_1483 | TFAP2C:MCF-7 |
| 483 | TCF3:set_2049 | 2225 | TCF3 | set_2049 | TCF3:Kasumi-1,RCH-ACV,GM12878 |
| 484 | TP73:set_2876 | 2220 | TP73 | set_2876 | TP73:SaOS-2 |
| 485 | MITF:set_2714 | 2220 | MITF | set_2714 | MITF:K-562,melanocyte |
| 486 | NR2F2:set_2935 | 2211 | NR2F2 | set_2935 | NR2F2:liver,K-562 |
| 487 | ETV1:set_1564 | 2210 | ETV1 | set_1564 | ETV1:LNCaP |
| 488 | CTCF:set_5027 | 2209 | CTCF | set_5027 | CTCF:THP-1 |
| 489 | ELF1:set_750 | 2204 | ELF1 | set_750 | ELF1:ME-1 |
| 490 | CEBPB:set_6960 | 2195 | CEBPB | set_6960 | CEBPB:IMR-90 |
| 491 | TBX21:set_3508 | 2194 | TBX21 | set_3508 | TBX21:TH1,GM12878 |
| 492 | ZNF263:set_1719 | 2193 | ZNF263 | set_1719 | ZNF263:HEK293T,HEK293,K-562 |
| 493 | TFAP2C:set_2661 | 2187 | TFAP2C | set_2661 | TFAP2C:SKBR3 |
| 494 | NFE2:set_3976 | 2186 | NFE2 | set_3976 | NFE2:ProEs,erythroid,K-562 |
| 495 | ZBTB7A:set_3090 | 2180 | ZBTB7A | set_3090 | ZBTB7A:Ishikawa,Hep-G2,HUDEP-2,K-562 |
| 496 | MYB:set_3355 | 2170 | MYB | set_3355 | MYB:CD4 |
| 497 | CEBPD:set_5042 | 2166 | CEBPD | set_5042 | CEBPD:HAEC |
| 498 | GATA2:set_1709 | 2161 | GATA2 | set_1709 | GATA2:ESF |
| 499 | ZBTB7A:set_692 | 2156 | ZBTB7A | set_692 | ZBTB7A:Ishikawa,K-562 |
| 500 | NR3C1:set_2370 | 2155 | NR3C1 | set_2370 | NR3C1:U2OS |
| 501 | JUND:set_6322 | 2148 | JUND | set_6322 | JUND:liver |
| 502 | USF2:set_999 | 2140 | USF2 | set_999 | USF2:HeLa-S3 |
| 503 | POU2F2:set_392 | 2127 | POU2F2 | set_392 | POU2F2:HBL1,GM12878,GM12891 |
| 504 | ZBTB7A:set_2292 | 2119 | ZBTB7A | set_2292 | ZBTB7A:HUDEP-2,K-562 |
| 505 | MAX:set_3677 | 2115 | MAX | set_3677 | MAX:Hep-G2 |
| 506 | FOS:set_1902 | 2109 | FOS | set_1902 | FOS:MNNG-HOS |
| 507 | RELA:set_4739 | 2093 | RELA | set_4739 | RELA:HUVEC-C |
| 508 | RBPJ:set_2376 | 2088 | RBPJ | set_2376 | RBPJ:NHEK |
| 509 | CREB1:set_3007 | 2080 | CREB1 | set_3007 | CREB1:MDA-MB-134-VI |
| 510 | USF1:set_2479 | 2078 | USF1 | set_2479 | USF1:Ishikawa |
| 511 | HSF1:set_1940 | 2074 | HSF1 | set_1940 | HSF1:NCI-H838 |
| 512 | JUND:set_941 | 2073 | JUND | set_941 | JUND:A-549 |
| 513 | YY1:set_2302 | 2072 | YY1 | set_2302 | YY1:HCT-116 |
| 514 | STAT1:set_1516 | 2062 | STAT1 | set_1516 | STAT1:NCI-H358 |
| 515 | KLF9:set_1727 | 2051 | KLF9 | set_1727 | KLF9:GBM1A,HEK293,MCF-7 |
| 516 | E2F1:set_5982 | 2047 | E2F1 | set_5982 | E2F1:K-562 |
| 517 | USF1:set_2498 | 2046 | USF1 | set_2498 | USF1:K-562 |
| 518 | EGR1:set_2140 | 2044 | EGR1 | set_2140 | EGR1:K-562,GM12878 |
| 519 | NRF1:set_1039 | 2042 | NRF1 | set_1039 | NRF1:HMEC-1 |
| 520 | JUN:set_4738 | 2034 | JUN | set_4738 | JUN:MCF-7 |
| 521 | ZBED1:set_3504 | 2031 | ZBED1 | set_3504 | ZBED1:GM12878 |
| 522 | EGR1:set_2872 | 2026 | EGR1 | set_2872 | EGR1:GM12878 |
| 523 | TFAP4:set_4270 | 2025 | TFAP4 | set_4270 | TFAP4:DLD-1 |
| 524 | TEAD1:set_4195 | 2010 | TEAD1 | set_4195 | TEAD1:MCF-7,T-47D |
| 525 | SMAD3:set_329 | 1997 | SMAD3 | set_329 | SMAD3:HCC1954,MDA-MB-231 |
| 526 | ZNF24:set_2237 | 1992 | ZNF24 | set_2237 | ZNF24:MCF-7 |
| 527 | STAT1:set_3078 | 1985 | STAT1 | set_3078 | STAT1:SET-2,CD14 |
| 528 | MYC:set_157 | 1982 | MYC | set_157 | MYC:U2OS |
| 529 | XBP1:set_2982 | 1980 | XBP1 | set_2982 | XBP1:HS578T,MDA-MB-231 |
| 530 | E2F1:set_3417 | 1969 | E2F1 | set_3417 | E2F1:LNCaP |
| 531 | ZBTB7A:set_61 | 1966 | ZBTB7A | set_61 | ZBTB7A:HEK293 |
| 532 | IRF1:set_2330 | 1958 | IRF1 | set_2330 | IRF1:monocyte |
| 533 | ONECUT1:set_10478 | 1957 | ONECUT1 | set_10478 | ONECUT1:H9 |
| 534 | RELB:set_292 | 1953 | RELB | set_292 | RELB:L1236 |
| 535 | TBX2:set_802 | 1952 | TBX2 | set_802 | TBX2:Kelly,SK-N-BE2-C |
| 536 | ZNF740:set_1769 | 1949 | ZNF740 | set_1769 | ZNF740:K-562 |
| 537 | ETV6:set_2869 | 1947 | ETV6 | set_2869 | ETV6:K-562 |
| 538 | RBPJ:set_4779 | 1943 | RBPJ | set_4779 | RBPJ:MUTUL |
| 539 | KLF5:set_507 | 1938 | KLF5 | set_507 | KLF5:HEK293,KATOIII |
| 540 | PKNOX1:set_934 | 1936 | PKNOX1 | set_934 | PKNOX1:MCF-7 |
| 541 | FOS:set_1150 | 1932 | FOS | set_1150 | FOS:IMR-90 |
| 542 | NEUROD1:set_1430 | 1929 | NEUROD1 | set_1430 | NEUROD1:K-562,D283-Med,D341-Med |
| 543 | PBX3:set_922 | 1928 | PBX3 | set_922 | PBX3:A-549 |
| 544 | ZBTB33:set_1199 | 1927 | ZBTB33 | set_1199 | ZBTB33:A-549 |
| 545 | TFAP2A:set_1153 | 1926 | TFAP2A | set_1153 | TFAP2A:WA09,MCF-7 |
| 546 | MXI1:set_1222 | 1923 | MXI1 | set_1222 | MXI1:HeLa-S3 |
| 547 | EGR1:set_5226 | 1922 | EGR1 | set_5226 | EGR1:liver |
| 548 | EBF1:set_3661 | 1913 | EBF1 | set_3661 | EBF1:MUTUL,GM12878 |
| 549 | ASCL1:set_3683 | 1907 | ASCL1 | set_3683 | ASCL1:SCLC |
| 550 | FOXK1:set_522 | 1906 | FOXK1 | set_522 | FOXK1:HEK293T |
| 551 | NR3C1:set_4282 | 1905 | NR3C1 | set_4282 | NR3C1:MCF-7 |
| 552 | MAX:set_3828 | 1905 | MAX | set_3828 | MAX:P493-6 |
| 553 | SMAD3:set_2785 | 1896 | SMAD3 | set_2785 | SMAD3:HCC1954,NCI-H441,MDA-MB-231 |
| 554 | IRF4:set_11342 | 1896 | IRF4 | set_11342 | IRF4:NCI-H929 |
| 555 | FLI1:set_150 | 1890 | FLI1 | set_150 | FLI1:UAE |
| 556 | HNF4G:set_4204 | 1881 | HNF4G | set_4204 | HNF4G:liver,Hep-G2 |
| 557 | CUX1:set_8491 | 1881 | CUX1 | set_8491 | CUX1:MCF-7 |
| 558 | ZBTB7A:set_4116 | 1874 | ZBTB7A | set_4116 | ZBTB7A:HUDEP-2 |
| 559 | PAX7:set_3847 | 1856 | PAX7 | set_3847 | PAX7:H9 |
| 560 | MAFK:set_2013 | 1856 | MAFK | set_2013 | MAFK:A-549 |
| 561 | ELF1:set_1701 | 1855 | ELF1 | set_1701 | ELF1:SK-N-MC |
| 562 | MEF2B:set_5140 | 1853 | MEF2B | set_5140 | MEF2B:GM12878,tonsil |
| 563 | MYCN:set_754 | 1844 | MYCN | set_754 | MYCN:Kelly,NGP |
| 564 | GATA6:set_5970 | 1842 | GATA6 | set_5970 | GATA6:YCC-3 |
| 565 | CTCF:set_2338 | 1840 | CTCF | set_2338 | CTCF:GM19239 |
| 566 | STAT3:set_5062 | 1835 | STAT3 | set_5062 | STAT3:MCF10A-Er-Src,MCF-10A |
| 567 | ERG:set_635 | 1833 | ERG | set_635 | ERG:CD34 |
| 568 | USF2:set_3723 | 1827 | USF2 | set_3723 | USF2:GM12878 |
| 569 | ETS1:set_3016 | 1824 | ETS1 | set_3016 | ETS1:Hep-G2 |
| 570 | MEIS1:set_10812 | 1823 | MEIS1 | set_10812 | MEIS1:CHRF28811 |
| 571 | POU5F1:set_3165 | 1822 | POU5F1 | set_3165 | POU5F1:BGO3,WA01 |
| 572 | MYCN:set_10319 | 1810 | MYCN | set_10319 | MYCN:RH4 |
| 573 | ESR1:set_2280 | 1804 | ESR1 | set_2280 | ESR1:Ishikawa,MCF-7 |
| 574 | ELF1:set_4601 | 1802 | ELF1 | set_4601 | ELF1:MCF-7 |
| 575 | RELA:set_3303 | 1799 | RELA | set_3303 | RELA:MCF-7 |
| 576 | ETS1:set_5089 | 1796 | ETS1 | set_5089 | ETS1:HEY-A8 |
| 577 | ASCL1:set_4220 | 1786 | ASCL1 | set_4220 | ASCL1:NCI-H889 |
| 578 | FOSL1:set_2506 | 1768 | FOSL1 | set_2506 | FOSL1:MNNG-HOS |
| 579 | USF1:set_7197 | 1765 | USF1 | set_7197 | USF1:A-549 |
| 580 | PKNOX1:set_1987 | 1753 | PKNOX1 | set_1987 | PKNOX1:HEK293T |
| 581 | TFDP1:set_3217 | 1746 | TFDP1 | set_3217 | TFDP1:K-562,MM1-S |
| 582 | NRF1:set_1016 | 1737 | NRF1 | set_1016 | NRF1:HCC1954 |
| 583 | GATA6:set_7514 | 1735 | GATA6 | set_7514 | GATA6:PATU8988,YCC-3 |
| 584 | CEBPA:set_3269 | 1731 | CEBPA | set_3269 | CEBPA:Hep-G2,Kasumi-1 |
| 585 | GATA2:set_1539 | 1731 | GATA2 | set_1539 | GATA2:ME-1 |
| 586 | ZNF263:set_3132 | 1724 | ZNF263 | set_3132 | ZNF263:HEK293T |
| 587 | TCF7L2:set_1411 | 1721 | TCF7L2 | set_1411 | TCF7L2:PANC-1 |
| 588 | CEBPB:set_2007 | 1706 | CEBPB | set_2007 | CEBPB:HL-60 |
| 589 | ATF7:set_3432 | 1700 | ATF7 | set_3432 | ATF7:K-562,GM12878 |
| 590 | NFATC3:set_3441 | 1691 | NFATC3 | set_3441 | NFATC3:Hep-G2,GM12878 |
| 591 | IRF1:set_2067 | 1690 | IRF1 | set_2067 | IRF1:PDAC,K-562 |
| 592 | JUND:set_1980 | 1688 | JUND | set_1980 | JUND:A-549,Hep-G2,K-562,WA01 |
| 593 | RBPJ:set_1409 | 1677 | RBPJ | set_1409 | RBPJ:Hep-G2,GSC8-11 |
| 594 | E2F6:set_4024 | 1675 | E2F6 | set_4024 | E2F6:K-562,A-549 |
| 595 | GATA2:set_1055 | 1675 | GATA2 | set_1055 | GATA2:SH-SY5Y |
| 596 | ZNF24:set_5424 | 1675 | ZNF24 | set_5424 | ZNF24:Hep-G2 |
| 597 | POU2F2:set_3497 | 1674 | POU2F2 | set_3497 | POU2F2:GM12878,GM12891 |
| 598 | STAT3:set_3287 | 1672 | STAT3 | set_3287 | STAT3:SU-DHL-2 |
| 599 | HNF1A:set_8704 | 1670 | HNF1A | set_8704 | HNF1A:NY8 |
| 600 | TEAD4:set_6526 | 1669 | TEAD4 | set_6526 | TEAD4:SK-N-BE2,BE2C |
| 601 | ERF:set_1566 | 1666 | ERF | set_1566 | ERF:VCaP |
| 602 | GRHL2:set_1431 | 1661 | GRHL2 | set_1431 | GRHL2:MCF-7-TAMR-1,MCF-7-WS8 |
| 603 | POU5F1:set_2418 | 1660 | POU5F1 | set_2418 | POU5F1:NCCIT |
| 604 | ZBTB7A:set_972 | 1654 | ZBTB7A | set_972 | ZBTB7A:Hep-G2,K-562 |
| 605 | PPARG:set_436 | 1653 | PPARG | set_436 | PPARG:Hep-G2,HUVEC-C |
| 606 | ELF4:set_3961 | 1653 | ELF4 | set_3961 | ELF4:HEK293T,K-562 |
| 607 | ZBTB7A:set_1376 | 1651 | ZBTB7A | set_1376 | ZBTB7A:HUDEP-2,HEK293,Hep-G2,K-562,Ishikawa |
| 608 | NR2F1:set_2398 | 1638 | NR2F1 | set_2398 | NR2F1:MCF-7,K-562 |
| 609 | ONECUT1:set_10005 | 1627 | ONECUT1 | set_10005 | ONECUT1:liver,H9 |
| 610 | MXI1:set_4393 | 1626 | MXI1 | set_4393 | MXI1:IMR-90,SK-N-SH |
| 611 | MYB:set_1033 | 1619 | MYB | set_1033 | MYB:Jurkat,MOLT-3 |
| 612 | CEBPA:set_5300 | 1613 | CEBPA | set_5300 | CEBPA:Hep-G2,Kasumi-1,MV4-11,SKH1 |
| 613 | MNT:set_1650 | 1611 | MNT | set_1650 | MNT:MCF-7,K-562 |
| 614 | NFIC:set_8061 | 1607 | NFIC | set_8061 | NFIC:Hep-G2 |
| 615 | KLF5:set_575 | 1605 | KLF5 | set_575 | KLF5:GP5D |
| 616 | EHF:set_4934 | 1602 | EHF | set_4934 | EHF:RWPE-1,primary-bronchial-epithelial |
| 617 | FOXA1:set_5596 | 1592 | FOXA1 | set_5596 | FOXA1:VCaP,LNCaP-abl,LNCaP |
| 618 | NFATC3:set_91 | 1592 | NFATC3 | set_91 | NFATC3:GM12878,K-562 |
| 619 | TBX5:set_7258 | 1570 | TBX5 | set_7258 | TBX5:G296S,cardiomyocyte |
| 620 | PKNOX1:set_4490 | 1569 | PKNOX1 | set_4490 | PKNOX1:GM12878,K-562 |
| 621 | ESR1:set_2362 | 1560 | ESR1 | set_2362 | ESR1:U2OS |
| 622 | SPI1:set_3068 | 1559 | SPI1 | set_3068 | SPI1:monocyte,macrophage |
| 623 | MYC:set_582 | 1557 | MYC | set_582 | MYC:NCI-H2171 |
| 624 | NFE2:set_396 | 1556 | NFE2 | set_396 | NFE2:ProEs,K-562 |
| 625 | MAX:set_1600 | 1554 | MAX | set_1600 | MAX:HeLa-S3 |
| 626 | MYCN:set_5203 | 1553 | MYCN | set_5203 | MYCN:MYCN-3 |
| 627 | CTCF:set_5050 | 1548 | CTCF | set_5050 | CTCF:GM12891 |
| 628 | E2F6:set_1705 | 1543 | E2F6 | set_1705 | E2F6:A-549 |
| 629 | ESR1:set_1310 | 1541 | ESR1 | set_1310 | ESR1:MSF-7 |
| 630 | JUND:set_2318 | 1537 | JUND | set_2318 | JUND:Hep-G2,K-562 |
| 631 | GRHL2:set_1335 | 1521 | GRHL2 | set_1335 | GRHL2:MCF-7-WS8 |
| 632 | TFAP2A:set_1884 | 1516 | TFAP2A | set_1884 | TFAP2A:WA09 |
| 633 | MEF2D:set_3648 | 1514 | MEF2D | set_3648 | MEF2D:K-562 |
| 634 | GATA2:set_3588 | 1510 | GATA2 | set_3588 | GATA2:LNCaP,SKH1,ME-1 |
| 635 | PBX2:set_1767 | 1504 | PBX2 | set_1767 | PBX2:Hep-G2,K-562 |
| 636 | PPARG:set_806 | 1502 | PPARG | set_806 | PPARG:HT29 |
| 637 | ATF7:set_3158 | 1495 | ATF7 | set_3158 | ATF7:GM12878,K-562 |
| 638 | SPIB:set_6426 | 1491 | SPIB | set_6426 | SPIB:OCI-Ly10,OCI-Ly3 |
| 639 | MAX:set_6739 | 1489 | MAX | set_6739 | MAX:liver |
| 640 | NR3C1:set_5126 | 1483 | NR3C1 | set_5126 | NR3C1:K-562 |
| 641 | ZNF143:set_1029 | 1479 | ZNF143 | set_1029 | ZNF143:FLP143HA,FLP76 |
| 642 | FOS:set_7783 | 1475 | FOS | set_7783 | FOS:endothelial |
| 643 | CEBPA:set_1241 | 1475 | CEBPA | set_1241 | CEBPA:Hep-G2,MV4-11,SKH1,Kasumi-1 |
| 644 | TBX21:set_6200 | 1474 | TBX21 | set_6200 | TBX21:CD4 |
| 645 | BACH2:set_6198 | 1470 | BACH2 | set_6198 | BACH2:B-cell |
| 646 | ETV1:set_1577 | 1469 | ETV1 | set_1577 | ETV1:MDA-Pca-2b,LNCaP |
| 647 | JUND:set_10919 | 1466 | JUND | set_10919 | JUND:SK-N-SH |
| 648 | VDR:set_4167 | 1466 | VDR | set_4167 | VDR:LCLGM10861 |
| 649 | TEAD4:set_1415 | 1463 | TEAD4 | set_1415 | TEAD4:Ishikawa,SK-MEL-147,K-562,WA01 |
| 650 | GATA3:set_962 | 1463 | GATA3 | set_962 | GATA3:A-549 |
| 651 | TP53:set_4030 | 1456 | TP53 | set_4030 | TP53:hESC |
| 652 | KLF5:set_2576 | 1454 | KLF5 | set_2576 | KLF5:GM12878 |
| 653 | YY1:set_3694 | 1452 | YY1 | set_3694 | YY1:Ishikawa |
| 654 | E2F6:set_2592 | 1445 | E2F6 | set_2592 | E2F6:K-562,HeLa-S3,A-549,WA01 |
| 655 | MAFF:set_4377 | 1444 | MAFF | set_4377 | MAFF:K-562,HeLa-S3,Hep-G2 |
| 656 | BHLHE40:set_1791 | 1443 | BHLHE40 | set_1791 | BHLHE40:GM12878,IMR-90 |
| 657 | ZNF263:set_4028 | 1438 | ZNF263 | set_4028 | ZNF263:HEK293T,HEK293 |
| 658 | JDP2:set_2737 | 1437 | JDP2 | set_2737 | JDP2:Loucy |
| 659 | IRF9:set_3650 | 1426 | IRF9 | set_3650 | IRF9:K-562 |
| 660 | HIF1A:set_8651 | 1423 | HIF1A | set_8651 | HIF1A:T-47D,U2OS |
| 661 | ETS1:set_1350 | 1417 | ETS1 | set_1350 | ETS1:786-O |
| 662 | POU2F2:set_8564 | 1415 | POU2F2 | set_8564 | POU2F2:HNPC |
| 663 | TBX2:set_1775 | 1407 | TBX2 | set_1775 | TBX2:SK-N-BE2-C |
| 664 | TCF7L2:set_353 | 1406 | TCF7L2 | set_353 | TCF7L2:HCT-116,PANC-1 |
| 665 | VDR:set_1108 | 1402 | VDR | set_1108 | VDR:LX2,THP-1 |
| 666 | ETS1:set_1920 | 1401 | ETS1 | set_1920 | ETS1:K-562,HUVEC-C |
| 667 | NFIA:set_5713 | 1398 | NFIA | set_5713 | NFIA:K-562 |
| 668 | NR2F2:set_2321 | 1397 | NR2F2 | set_2321 | NR2F2:K-562,MCF-7 |
| 669 | SMAD3:set_3876 | 1396 | SMAD3 | set_3876 | SMAD3:BGO3,MDA-MB-231 |
| 670 | TFAP2C:set_8100 | 1392 | TFAP2C | set_8100 | TFAP2C:A-375,MDA-MB-453,BT-474,MCF-7,SKBR3 |
| 671 | PPARG:set_6295 | 1390 | PPARG | set_6295 | PPARG:ASC |
| 672 | EBF1:set_2987 | 1389 | EBF1 | set_2987 | EBF1:GM12878,MUTUL |
| 673 | GRHL2:set_789 | 1388 | GRHL2 | set_789 | GRHL2:OVCAR-3 |
| 674 | RBPJ:set_10132 | 1385 | RBPJ | set_10132 | RBPJ:LCL |
| 675 | GRHL2:set_6205 | 1378 | GRHL2 | set_6205 | GRHL2:MCF-7,MCF-7-TAMR-1 |
| 676 | E2F1:set_4417 | 1376 | E2F1 | set_4417 | E2F1:MCF-7 |
| 677 | NEUROD1:set_1758 | 1371 | NEUROD1 | set_1758 | NEUROD1:K-562,D341-Med |
| 678 | ZBTB7A:set_2238 | 1369 | ZBTB7A | set_2238 | ZBTB7A:Ishikawa,Hep-G2,K-562 |
| 679 | RELA:set_4785 | 1366 | RELA | set_4785 | RELA:KB |
| 680 | CEBPA:set_5415 | 1365 | CEBPA | set_5415 | CEBPA:Hep-G2,liver |
| 681 | STAT3:set_1713 | 1364 | STAT3 | set_1713 | STAT3:MDA-MB-157 |
| 682 | ELK1:set_3116 | 1363 | ELK1 | set_3116 | ELK1:K-562 |
| 683 | PAX5:set_4433 | 1363 | PAX5 | set_4433 | PAX5:GM12891,GM12878,GM12892 |
| 684 | CEBPB:set_9308 | 1360 | CEBPB | set_9308 | CEBPB:SUM159PT |
| 685 | ZEB1:set_1628 | 1356 | ZEB1 | set_1628 | ZEB1:RKO |
| 686 | YY2:set_3049 | 1355 | YY2 | set_3049 | YY2:HeLa |
| 687 | ETV6:set_4978 | 1353 | ETV6 | set_4978 | ETV6:K-562,GM12878 |
| 688 | NFIC:set_2588 | 1350 | NFIC | set_2588 | NFIC:Ishikawa,K-562 |
| 689 | PKNOX1:set_3429 | 1346 | PKNOX1 | set_3429 | PKNOX1:MCF-7,GM12878,K-562 |
| 690 | FOSL1:set_2921 | 1345 | FOSL1 | set_2921 | FOSL1:143B |
| 691 | HOXB13:set_1047 | 1345 | HOXB13 | set_1047 | HOXB13:G-401 |
| 692 | TEAD4:set_2214 | 1342 | TEAD4 | set_2214 | TEAD4:K-562,WA01 |
| 693 | ESR1:set_11231 | 1341 | ESR1 | set_11231 | ESR1:T-47D |
| 694 | TFDP1:set_3986 | 1339 | TFDP1 | set_3986 | TFDP1:U266B1,K-562,MM1-S |
| 695 | MXI1:set_3123 | 1338 | MXI1 | set_3123 | MXI1:Hep-G2 |
| 696 | MAX:set_4588 | 1337 | MAX | set_4588 | MAX:MCF-7 |
| 697 | SRF:set_4923 | 1335 | SRF | set_4923 | SRF:Hep-G2 |
| 698 | YY1:set_2251 | 1334 | YY1 | set_2251 | YY1:Hep-G2 |
| 699 | RFX5:set_2959 | 1332 | RFX5 | set_2959 | RFX5:HeLa-S3,Hep-G2 |
| 700 | KLF4:set_2687 | 1332 | KLF4 | set_2687 | KLF4:keratinocyte,PDAC |
| 701 | MYCN:set_1732 | 1332 | MYCN | set_1732 | MYCN:Kelly,SHEP-21N |
| 702 | NR3C1:set_2478 | 1330 | NR3C1 | set_2478 | NR3C1:A-549,Ishikawa |
| 703 | TEAD1:set_4549 | 1323 | TEAD1 | set_4549 | TEAD1:Hep-G2,MCF-7,T-47D |
| 704 | JUN:set_2131 | 1305 | JUN | set_2131 | JUN:K-562,HAEC |
| 705 | MXI1:set_1138 | 1305 | MXI1 | set_1138 | MXI1:neural |
| 706 | RELA:set_3327 | 1303 | RELA | set_3327 | RELA:SW480 |
| 707 | ESR1:set_7801 | 1302 | ESR1 | set_7801 | ESR1:endometrioid-adenocarcinoma |
| 708 | TEAD1:set_5612 | 1301 | TEAD1 | set_5612 | TEAD1:Hep-G2,MCF-7 |
| 709 | MYC:set_3875 | 1301 | MYC | set_3875 | MYC:MCF-10A |
| 710 | FOXP1:set_1218 | 1295 | FOXP1 | set_1218 | FOXP1:Hep-G2,H9,VCaP |
| 711 | FOXK2:set_4977 | 1293 | FOXK2 | set_4977 | FOXK2:Hep-G2,K-562 |
| 712 | ERG:set_4894 | 1292 | ERG | set_4894 | ERG:MCF-7 |
| 713 | REST:set_47 | 1288 | REST | set_47 | REST:HEK293 |
| 714 | ESR1:set_5044 | 1281 | ESR1 | set_5044 | ESR1:MDA-MB-134-VI |
| 715 | CTCF:set_2807 | 1277 | CTCF | set_2807 | CTCF:SUM159 |
| 716 | MNT:set_1283 | 1276 | MNT | set_1283 | MNT:Hep-G2,K-562 |
| 717 | TFAP4:set_6827 | 1271 | TFAP4 | set_6827 | TFAP4:Kasumi-1 |
| 718 | MEF2C:set_2960 | 1267 | MEF2C | set_2960 | MEF2C:MOLM-13,GM12878 |
| 719 | ASCL1:set_1050 | 1265 | ASCL1 | set_1050 | ASCL1:NCI-H2107,SCLC,NCI-H889 |
| 720 | BHLHE40:set_1394 | 1264 | BHLHE40 | set_1394 | BHLHE40:GM12878,IMR-90,K-562 |
| 721 | HSF1:set_427 | 1260 | HSF1 | set_427 | HSF1:MO91,NCI-H838,451Lu |
| 722 | RELA:set_1759 | 1255 | RELA | set_1759 | RELA:BJAB |
| 723 | GATA2:set_5950 | 1255 | GATA2 | set_5950 | GATA2:TF1 |
| 724 | LHX2:set_2004 | 1253 | LHX2 | set_2004 | LHX2:retina |
| 725 | JUN:set_5066 | 1252 | JUN | set_5066 | JUN:MCF10A-Er-Src,HAEC |
| 726 | ETV1:set_3185 | 1251 | ETV1 | set_3185 | ETV1:GIST |
| 727 | SPI1:set_5481 | 1250 | SPI1 | set_5481 | SPI1:K-562 |
| 728 | NFKB1:set_3493 | 1249 | NFKB1 | set_3493 | NFKB1:MCF10A-Er-Src,L1236 |
| 729 | REST:set_4432 | 1248 | REST | set_4432 | REST:CD4 |
| 730 | CTCFL:set_1461 | 1243 | CTCFL | set_1461 | CTCFL:delta-47,K-562 |
| 731 | ZEB1:set_2134 | 1238 | ZEB1 | set_2134 | ZEB1:GM12878,neuron |
| 732 | IRF3:set_3219 | 1236 | IRF3 | set_3219 | IRF3:GM12878 |
| 733 | NR2F2:set_5310 | 1231 | NR2F2 | set_5310 | NR2F2:liver,K-562,MCF-7 |
| 734 | SIX2:set_4652 | 1228 | SIX2 | set_4652 | SIX2:HEK |
| 735 | SPI1:set_1580 | 1227 | SPI1 | set_1580 | SPI1:ME-1 |
| 736 | NR2F2:set_5392 | 1227 | NR2F2 | set_5392 | NR2F2:Hep-G2 |
| 737 | MAFK:set_2779 | 1226 | MAFK | set_2779 | MAFK:HeLa-S3 |
| 738 | RELA:set_5204 | 1223 | RELA | set_5204 | RELA:HAEC,HUVEC-C |
| 739 | NFKB1:set_5007 | 1223 | NFKB1 | set_5007 | NFKB1:L1236,MCF10A-Er-Src |
| 740 | SOX4:set_5461 | 1223 | SOX4 | set_5461 | SOX4:HCC1954 |
| 741 | ERG:set_3551 | 1218 | ERG | set_3551 | ERG:AMLPZ12 |
| 742 | ESR1:set_3194 | 1216 | ESR1 | set_3194 | ESR1:Ishikawa,breast,MCF-7 |
| 743 | ETV1:set_3476 | 1215 | ETV1 | set_3476 | ETV1:COLO-800 |
| 744 | SMAD3:set_1711 | 1204 | SMAD3 | set_1711 | SMAD3:NCI-H441,MDA-MB-231 |
| 745 | ETS1:set_2792 | 1198 | ETS1 | set_2792 | ETS1:OVCAR-8 |
| 746 | ATF7:set_3740 | 1197 | ATF7 | set_3740 | ATF7:Hep-G2 |
| 747 | GATA3:set_5439 | 1191 | GATA3 | set_5439 | GATA3:breast,MCF-7,T-47D |
| 748 | NR3C1:set_6149 | 1188 | NR3C1 | set_6149 | NR3C1:GM12878 |
| 749 | KLF9:set_2805 | 1188 | KLF9 | set_2805 | KLF9:GBM1A,MCF-7 |
| 750 | ZEB1:set_5573 | 1188 | ZEB1 | set_5573 | ZEB1:Hep-G2 |
| 751 | TBX5:set_2490 | 1187 | TBX5 | set_2490 | TBX5:cardiomyocyte,G296S |
| 752 | GRHL2:set_4222 | 1187 | GRHL2 | set_4222 | GRHL2:LNCaP |
| 753 | TEAD1:set_9523 | 1183 | TEAD1 | set_9523 | TEAD1:HUCCT1 |
| 754 | IRF5:set_4993 | 1182 | IRF5 | set_4993 | IRF5:GM12878 |
| 755 | NR2F1:set_1017 | 1180 | NR2F1 | set_1017 | NR2F1:MCF-7,K-562,GM12878 |
| 756 | BHLHE40:set_1677 | 1176 | BHLHE40 | set_1677 | BHLHE40:GM12878,K-562 |
| 757 | NFIC:set_1203 | 1176 | NFIC | set_1203 | NFIC:Ishikawa,SK-N-SH |
| 758 | TP53:set_1460 | 1171 | TP53 | set_1460 | TP53:MCF-7 |
| 759 | ELK1:set_7872 | 1169 | ELK1 | set_7872 | ELK1:MCF-7 |
| 760 | STAT3:set_7864 | 1163 | STAT3 | set_7864 | STAT3:MDA-MB-231 |
| 761 | FOS:set_7956 | 1147 | FOS | set_7956 | FOS:MCF-7 |
| 762 | FOXP1:set_3159 | 1147 | FOXP1 | set_3159 | FOXP1:H9,VCaP |
| 763 | HNF4G:set_7914 | 1144 | HNF4G | set_7914 | HNF4G:liver |
| 764 | MAFK:set_7709 | 1140 | MAFK | set_7709 | MAFK:IMR-90,Hep-G2,A-549 |
| 765 | MAX:set_3989 | 1136 | MAX | set_3989 | MAX:SK-N-SH |
| 766 | TEAD4:set_2396 | 1134 | TEAD4 | set_2396 | TEAD4:Ishikawa,WA01 |
| 767 | ZBED1:set_6246 | 1128 | ZBED1 | set_6246 | ZBED1:K-562,GM12878 |
| 768 | GRHL2:set_2660 | 1128 | GRHL2 | set_2660 | GRHL2:OVCA429 |
| 769 | NFIC:set_1803 | 1128 | NFIC | set_1803 | NFIC:Ishikawa,SK-N-SH,K-562 |
| 770 | ZBTB7A:set_1643 | 1124 | ZBTB7A | set_1643 | ZBTB7A:Ishikawa,HUDEP-2,K-562 |
| 771 | IRF4:set_6237 | 1122 | IRF4 | set_6237 | IRF4:GM12878,NCI-H929,MM1-S |
| 772 | USF1:set_3328 | 1118 | USF1 | set_3328 | USF1:Hep-G2 |
| 773 | NRF1:set_5567 | 1116 | NRF1 | set_5567 | NRF1:HMEC-1,HCC1954 |
| 774 | USF1:set_2752 | 1113 | USF1 | set_2752 | USF1:GM12878,WA01,K-562,HCT-116,Ishikawa,SK-N-SH,Hep-G2,A-549 |
| 775 | NR3C1:set_6944 | 1102 | NR3C1 | set_6944 | NR3C1:HASM2,IMR-90 |
| 776 | FOXP1:set_2657 | 1098 | FOXP1 | set_2657 | FOXP1:Hep-G2,VCaP |
| 777 | ASCL1:set_1972 | 1095 | ASCL1 | set_1972 | ASCL1:NCI-H2107,SCLC |
| 778 | CTCFL:set_1599 | 1091 | CTCFL | set_1599 | CTCFL:delta-47 |
| 779 | TCF3:set_4228 | 1089 | TCF3 | set_4228 | TCF3:Kasumi-1,RCH-ACV |
| 780 | SP1:set_3318 | 1084 | SP1 | set_3318 | SP1:liver,Hep-G2 |
| 781 | HINFP:set_1642 | 1084 | HINFP | set_1642 | HINFP:K-562 |
| 782 | KLF16:set_1078 | 1082 | KLF16 | set_1078 | KLF16:K-562,HEK293 |
| 783 | ELF1:set_3106 | 1080 | ELF1 | set_3106 | ELF1:A-549,ME-1,SK-N-MC,Hep-G2,K-562,SK-N-SH,GM12878,MCF-7 |
| 784 | STAT3:set_2434 | 1078 | STAT3 | set_2434 | STAT3:NCI-H358 |
| 785 | YY1:set_4383 | 1076 | YY1 | set_4383 | YY1:A-549 |
| 786 | CEBPA:set_2944 | 1074 | CEBPA | set_2944 | CEBPA:Hep-G2,MV4-11 |
| 787 | STAT1:set_1877 | 1074 | STAT1 | set_1877 | STAT1:HeLa-S3 |
| 788 | YY1:set_4326 | 1073 | YY1 | set_4326 | YY1:SK-N-SH |
| 789 | IRF1:set_7998 | 1071 | IRF1 | set_7998 | IRF1:HAEC |
| 790 | TFAP4:set_5011 | 1068 | TFAP4 | set_5011 | TFAP4:LNCaP |
| 791 | RELA:set_1021 | 1066 | RELA | set_1021 | RELA:HEK293,monocyte |
| 792 | ZEB1:set_5292 | 1066 | ZEB1 | set_5292 | ZEB1:GM12878,HEK293 |
| 793 | CEBPA:set_6321 | 1063 | CEBPA | set_6321 | CEBPA:liver |
| 794 | RELA:set_3272 | 1062 | RELA | set_3272 | RELA:HDF |
| 795 | TEAD4:set_3804 | 1060 | TEAD4 | set_3804 | TEAD4:SK-MEL-147,K-562 |
| 796 | POU2F2:set_5671 | 1056 | POU2F2 | set_5671 | POU2F2:GM12891 |
| 797 | JUNB:set_278 | 1055 | JUNB | set_278 | JUNB:MCF10A-Er-Src,HAEC |
| 798 | ETS1:set_955 | 1053 | ETS1 | set_955 | ETS1:A-549 |
| 799 | FOXP1:set_5262 | 1052 | FOXP1 | set_5262 | FOXP1:Hep-G2,VCaP,H9 |
| 800 | TFAP2C:set_3774 | 1048 | TFAP2C | set_3774 | TFAP2C:UCLA1-hESCs,MDA-MB-453,BT-474,MCF-7,SKBR3 |
| 801 | MYCN:set_4908 | 1044 | MYCN | set_4908 | MYCN:Kelly,SHEP-21N,NGP |
| 802 | MYB:set_2970 | 1043 | MYB | set_2970 | MYB:Jurkat,MOLT-3,CD4,THP-1 |
| 803 | FOSL2:set_4164 | 1042 | FOSL2 | set_4164 | FOSL2:A-549,SK-MEL-147 |
| 804 | TFAP4:set_3448 | 1040 | TFAP4 | set_3448 | TFAP4:DLD-1,Hep-G2 |
| 805 | PAX5:set_6725 | 1039 | PAX5 | set_6725 | PAX5:GM12878,GM12891 |
| 806 | E2F6:set_4870 | 1037 | E2F6 | set_4870 | E2F6:A-549,WA01 |
| 807 | KLF4:set_501 | 1037 | KLF4 | set_501 | KLF4:BJ,keratinocyte |
| 808 | MYC:set_3883 | 1035 | MYC | set_3883 | MYC:GP5D |
| 809 | ZNF410:set_1452 | 1033 | ZNF410 | set_1452 | ZNF410:K-562 |
| 810 | PAX5:set_3782 | 1033 | PAX5 | set_3782 | PAX5:GM12891 |
| 811 | TFAP4:set_2473 | 1030 | TFAP4 | set_2473 | TFAP4:LNCaP,Hep-G2 |
| 812 | NR3C1:set_1602 | 1028 | NR3C1 | set_1602 | NR3C1:A-549,HASM2 |
| 813 | CREB1:set_3741 | 1028 | CREB1 | set_3741 | CREB1:MCF-7,Hep-G2 |
| 814 | FOXA1:set_3340 | 1027 | FOXA1 | set_3340 | FOXA1:liver |
| 815 | TEAD4:set_2474 | 1024 | TEAD4 | set_2474 | TEAD4:Ishikawa,K-562 |
| 816 | KLF9:set_4684 | 1023 | KLF9 | set_4684 | KLF9:GBM1A,MCF-7,HEK293 |
| 817 | RELA:set_7843 | 1022 | RELA | set_7843 | RELA:K-562 |
| 818 | PPARG:set_3521 | 1021 | PPARG | set_3521 | PPARG:Hep-G2,HT29 |
| 819 | ESR1:set_3689 | 1021 | ESR1 | set_3689 | ESR1:MSF-7,breast,MCF-7 |
| 820 | ETS1:set_900 | 1020 | ETS1 | set_900 | ETS1:HUVEC-C,786-O |
| 821 | TP53:set_990 | 1020 | TP53 | set_990 | TP53:GM06170 |
| 822 | MYCN:set_3143 | 1017 | MYCN | set_3143 | MYCN:Kelly,BE2C,NGP,SK-N-BE2-C |
| 823 | BCL6B:set_63 | 1016 | BCL6B | set_63 | BCL6B:HEK293 |
| 824 | CLOCK:set_6031 | 1015 | CLOCK | set_6031 | CLOCK:BA40-neocortex,BA10-neocortex |
| 825 | POU5F1:set_1380 | 1015 | POU5F1 | set_1380 | POU5F1:BJ1-hTERT,WA01 |
| 826 | TEAD4:set_4044 | 1010 | TEAD4 | set_4044 | TEAD4:SNU-216 |
| 827 | EBF1:set_4953 | 1009 | EBF1 | set_4953 | EBF1:MUTUL,LCL,GM12878 |
| 828 | SPI1:set_3419 | 1008 | SPI1 | set_3419 | SPI1:CD34 |
| 829 | SRF:set_213 | 1001 | SRF | set_213 | SRF:K-562 |
