# Supplementary material for: Identification of upstream transcription factor binding sites in orthologous genes using mixed Student’s t-test statistics
Source: PLoS Comput Biol. 2022 Jun 7;18(6):e1009773. doi: 10.1371/journal.pcbi.1009773 (PMC9205514; doi:10.1371/journal.pcbi.1009773)
Supplement: S3 Table — (DOCX) [file pcbi.1009773.s004.docx]

S3_Table. Detailed information of the publicly available Chip-Seq datasets (Altas-2021) with literature support

Table S3. Information of the ChIP-Atlas datasets

| # ID | Name | Species | Class |
| --- | --- | --- | --- |
| MA0003.1 | TFAP2A | Homo sapiens | Basic helix-span-helix factors (bHSH) |
| MA0014.2 | PAX5 | Homo sapiens | Paired box factors |
| MA0017.1 | NR2F1 | Homo sapiens | Nuclear receptors with C4 zinc fingers |
| MA0018.1 | CREB1 | Homo sapiens | Basic leucine zipper factors (bZIP) |
| MA0018.2 | CREB1 | Mus musculus Rattus norvegicus Homo sapiens | Basic leucine zipper factors (bZIP) |
| MA0018.3 | CREB1 | Homo sapiens | Basic leucine zipper factors (bZIP) |
| MA0024.1 | E2F1 | Homo sapiens | Fork head / winged helix factors |
| MA0024.2 | E2F1 | Homo sapiens | Fork head / winged helix factors |
| MA0024.3 | E2F1 | Homo sapiens | Fork head / winged helix factors |
| MA0028.1 | ELK1 | Homo sapiens | Tryptophan cluster factors |
| MA0028.2 | ELK1 | Homo sapiens | Tryptophan cluster factors |
| MA0030.1 | FOXF2 | Homo sapiens | Fork head / winged helix factors |
| MA0036.1 | GATA2 | Homo sapiens | Other C4 zinc finger-type factors |
| MA0036.2 | GATA2 | Homo sapiens | Other C4 zinc finger-type factors |
| MA0036.3 | GATA2 | Homo sapiens | Other C4 zinc finger-type factors |
| MA0037.1 | GATA3 | Homo sapiens | Other C4 zinc finger-type factors |
| MA0037.2 | GATA3 | Homo sapiens | Other C4 zinc finger-type factors |
| MA0037.3 | GATA3 | Homo sapiens | Other C4 zinc finger-type factors |
| MA0039.3 | KLF4 | Homo sapiens | C2H2 zinc finger factors |
| MA0046.2 | HNF1A | Homo sapiens | Homeo domain factors |
| MA0048.1 | NHLH1 | Homo sapiens | Basic helix-loop-helix factors (bHLH) |
| MA0051.1 | IRF2 | Homo sapiens | Tryptophan cluster factors |
| MA0052.1 | MEF2A | Homo sapiens | MADS box factors |
| MA0058.1 | MAX | Homo sapiens | Basic helix-loop-helix factors (bHLH) |
| MA0060.1 | NFYA | Mus musculus Rattus norvegicus Xenopus laevis Xenopus (Silurana) tropicalis Gallus gallus Homo sapiens Bos taurus Oryctolagus cuniculus | Other alpha |
| MA0060.2 | NFYA | Homo sapiens | Other alpha |
| MA0060.3 | NFYA | Homo sapiens | Other alpha |
| MA0062.1 | GABPA | Homo sapiens | Tryptophan cluster factors |
| MA0066.1 | PPARG | Homo sapiens | Nuclear receptors with C4 zinc fingers |
| MA0069.1 | Pax6 | Homo sapiens | Paired box factors |
| MA0073.1 | RREB1 | Homo sapiens | C2H2 zinc finger factors |
| MA0076.1 | ELK4 | Homo sapiens | Tryptophan cluster factors |
| MA0076.2 | ELK4 | Homo sapiens | Tryptophan cluster factors |
| MA0077.1 | SOX9 | Homo sapiens | High-mobility group (HMG) domain factors |
| MA0079.1 | SP1 | Homo sapiens | C2H2 zinc finger factors |
| MA0079.2 | SP1 | Mus musculus Rattus norvegicus Homo sapiens | C2H2 zinc finger factors |
| MA0079.3 | SP1 | Homo sapiens | C2H2 zinc finger factors |
| MA0080.1 | SPI1 | Homo sapiens | Tryptophan cluster factors |
| MA0080.2 | SPI1 | Homo sapiens | Tryptophan cluster factors |
| MA0080.4 | SPI1 | Homo sapiens | Tryptophan cluster factors |
| MA0083.1 | SRF | Homo sapiens | MADS box factors |
| MA0083.2 | SRF | Homo sapiens | MADS box factors |
| MA0083.3 | SRF | Homo sapiens | MADS box factors |
| MA0090.1 | TEAD1 | Homo sapiens | TEA domain factors |
| MA0090.2 | TEAD1 | Homo sapiens | TEA domain factors |
| MA0093.1 | USF1 | Homo sapiens | Basic helix-loop-helix factors (bHLH) |
| MA0093.2 | USF1 | Homo sapiens | Basic helix-loop-helix factors (bHLH) |
| MA0095.1 | YY1 | Homo sapiens | C2H2 zinc finger factors |
| MA0095.2 | YY1 | Homo sapiens | C2H2 zinc finger factors |
| MA0098.1 | ETS1 | Homo sapiens | Tryptophan cluster factors |
| MA0098.3 | ETS1 | Homo sapiens | Tryptophan cluster factors |
| MA0099.2 | FOS::JUN | Mus musculus Rattus norvegicus Homo sapiens | Basic leucine zipper factors (bZIP)::Basic leucine zipper factors (bZIP) |
| MA0099.3 | FOS::JUN | Homo sapiens | Basic leucine zipper factors (bZIP)::Basic leucine zipper factors (bZIP) |
| MA0100.3 | MYB | Homo sapiens | Tryptophan cluster factors |
| MA0101.1 | REL | Homo sapiens | Rel homology region (RHR) factors |
| MA0102.3 | CEBPA | Homo sapiens | Basic leucine zipper factors (bZIP) |
| MA0103.2 | ZEB1 | Homo sapiens | Homeo domain factors |
| MA0103.3 | ZEB1 | Homo sapiens | Homeo domain factors |
| MA0104.4 | MYCN | Homo sapiens | Basic helix-loop-helix factors (bHLH) |
| MA0105.1 | NFKB1 | Mus musculus Rattus norvegicus Homo sapiens Oryctolagus cuniculus | Rel homology region (RHR) factors |
| MA0105.2 | NFKB1 | Homo sapiens | Rel homology region (RHR) factors |
| MA0105.3 | NFKB1 | Homo sapiens | Rel homology region (RHR) factors |
| MA0105.4 | NFKB1 | Homo sapiens | Rel homology region (RHR) factors |
| MA0107.1 | RELA | Homo sapiens | Rel homology region (RHR) factors |
| MA0113.1 | NR3C1 | Mus musculus Rattus norvegicus Oncorhynchus mykiss Gallus gallus Homo sapiens | Nuclear receptors with C4 zinc fingers |
| MA0113.3 | NR3C1 | Homo sapiens | Nuclear receptors with C4 zinc fingers |
| MA0114.2 | HNF4A | Homo sapiens | Nuclear receptors with C4 zinc fingers |
| MA0124.1 | NKX3-1 | Homo sapiens | Homeo domain factors |
| MA0132.2 | PDX1 | Homo sapiens | Homeo domain factors |
| MA0137.1 | STAT1 | Homo sapiens | STAT domain factors |
| MA0137.2 | STAT1 | Homo sapiens | STAT domain factors |
| MA0137.3 | STAT1 | Homo sapiens | STAT domain factors |
| MA0138.1 | REST | Homo sapiens | C2H2 zinc finger factors |
| MA0138.2 | REST | Homo sapiens | C2H2 zinc finger factors |
| MA0140.2 | GATA1::TAL1 | Homo sapiens | Other C4 zinc finger-type factors::Basic helix-loop-helix factors (bHLH) |
| MA0147.3 | MYC | Homo sapiens | Basic helix-loop-helix factors (bHLH) |
| MA0148.1 | FOXA1 | Homo sapiens | Fork head / winged helix factors |
| MA0148.2 | FOXA1 | Homo sapiens | Fork head / winged helix factors |
| MA0148.3 | FOXA1 | Homo sapiens | Fork head / winged helix factors |
| MA0149.1 | EWSR1-FLI1 | Homo sapiens | Tryptophan cluster factors |
| MA0150.1 | NFE2L2 | Homo sapiens | Basic leucine zipper factors (bZIP) |
| MA0152.1 | NFATC2 | Mus musculus Rattus norvegicus Homo sapiens | Rel homology region (RHR) factors |
| MA0153.1 | HNF1B | Mus musculus Homo sapiens | Homeo domain factors |
| MA0153.2 | HNF1B | Homo sapiens | Homeo domain factors |
| MA0158.1 | HOXA5 | Mus musculus Homo sapiens | Homeo domain factors |
| MA0160.1 | NR4A2 | Mus musculus Rattus norvegicus Homo sapiens | Nuclear receptors with C4 zinc fingers |
| MA0161.1 | NFIC | Homo sapiens | SMAD/NF-1 DNA-binding domain factors |
| MA0161.2 | NFIC | Homo sapiens | SMAD/NF-1 DNA-binding domain factors |
| MA0162.2 | EGR1 | Homo sapiens | C2H2 zinc finger factors |
| MA0162.3 | EGR1 | Homo sapiens | C2H2 zinc finger factors |
| MA0163.1 | PLAG1 | Homo sapiens | C2H2 zinc finger factors |
| MA0258.1 | ESR2 | Homo sapiens | Nuclear receptors with C4 zinc fingers |
| MA0258.2 | ESR2 | Homo sapiens | Nuclear receptors with C4 zinc fingers |
| MA0259.1 | ARNT::HIF1A | Mus musculus Rattus rattus Homo sapiens Oryctolagus cuniculus | Basic helix-loop-helix factors (bHLH)::Basic helix-loop-helix factors (bHLH) |
| MA0442.1 | SOX10 | Mus musculus Rattus norvegicus Homo sapiens | High-mobility group (HMG) domain factors |
| MA0442.2 | SOX10 | Homo sapiens | High-mobility group (HMG) domain factors |
| MA0462.1 | BATF::JUN | Homo sapiens | Basic leucine zipper factors (bZIP)::Basic leucine zipper factors (bZIP) |
| MA0464.2 | BHLHE40 | Homo sapiens | Basic helix-loop-helix factors (bHLH) |
| MA0465.1 | CDX2 | Homo sapiens | Homeo domain factors |
| MA0466.1 | CEBPB | Homo sapiens | Basic leucine zipper factors (bZIP) |
| MA0466.2 | CEBPB | Homo sapiens | Basic leucine zipper factors (bZIP) |
| MA0468.1 | DUX4 | Homo sapiens | Homeo domain factors |
| MA0469.2 | E2F3 | Homo sapiens | Fork head / winged helix factors |
| MA0470.1 | E2F4 | Homo sapiens | Fork head / winged helix factors |
| MA0471.1 | E2F6 | Homo sapiens | Fork head / winged helix factors |
| MA0472.2 | EGR2 | Homo sapiens | C2H2 zinc finger factors |
| MA0473.1 | ELF1 | Homo sapiens | Tryptophan cluster factors |
| MA0473.2 | ELF1 | Homo sapiens | Tryptophan cluster factors |
| MA0475.1 | FLI1 | Homo sapiens | Tryptophan cluster factors |
| MA0475.2 | FLI1 | Homo sapiens | Tryptophan cluster factors |
| MA0476.1 | FOS | Homo sapiens | Basic leucine zipper factors (bZIP) |
| MA0477.1 | FOSL1 | Homo sapiens | Basic leucine zipper factors (bZIP) |
| MA0481.1 | FOXP1 | Homo sapiens | Fork head / winged helix factors |
| MA0481.2 | FOXP1 | Homo sapiens | Fork head / winged helix factors |
| MA0484.1 | HNF4G | Homo sapiens | Nuclear receptors with C4 zinc fingers |
| MA0486.1 | HSF1 | Homo sapiens | Heat shock factors |
| MA0486.2 | HSF1 | Homo sapiens | Heat shock factors |
| MA0488.1 | JUN | Homo sapiens | Basic leucine zipper factors (bZIP) |
| MA0489.1 | JUN(var.2) | Homo sapiens | Basic leucine zipper factors (bZIP) |
| MA0490.1 | JUNB | Homo sapiens | Basic leucine zipper factors (bZIP) |
| MA0491.1 | JUND | Homo sapiens | Basic leucine zipper factors (bZIP) |
| MA0492.1 | JUND(var.2) | Homo sapiens | Basic leucine zipper factors (bZIP) |
| MA0495.1 | MAFF | Homo sapiens | Basic leucine zipper factors (bZIP) |
| MA0495.2 | MAFF | Homo sapiens | Basic leucine zipper factors (bZIP) |
| MA0496.1 | MAFK | Homo sapiens | Basic leucine zipper factors (bZIP) |
| MA0496.2 | MAFK | Homo sapiens | Basic leucine zipper factors (bZIP) |
| MA0497.1 | MEF2C | Homo sapiens | MADS box factors |
| MA0498.2 | MEIS1 | Homo sapiens | Homeo domain factors |
| MA0501.1 | MAF::NFE2 | Homo sapiens | Basic leucine zipper factors (bZIP)::Basic leucine zipper factors (bZIP) |
| MA0502.1 | NFYB | Homo sapiens | Other alpha |
| MA0504.1 | NR2C2 | Homo sapiens | Nuclear receptors with C4 zinc fingers |
| MA0506.1 | NRF1 | Homo sapiens | Basic leucine zipper factors (bZIP) |
| MA0507.1 | POU2F2 | Homo sapiens | Homeo domain factors |
| MA0510.1 | RFX5 | Homo sapiens | Fork head / winged helix factors |
| MA0510.2 | RFX5 | Homo sapiens | Fork head / winged helix factors |
| MA0511.1 | RUNX2 | Homo sapiens | Runt domain factors |
| MA0511.2 | RUNX2 | Homo sapiens | Runt domain factors |
| MA0516.1 | SP2 | Homo sapiens | C2H2 zinc finger factors |
| MA0522.2 | TCF3 | Homo sapiens | Basic helix-loop-helix factors (bHLH) |
| MA0523.1 | TCF7L2 | Homo sapiens | High-mobility group (HMG) domain factors |
| MA0524.1 | TFAP2C | Homo sapiens | Basic helix-span-helix factors (bHSH) |
| MA0524.2 | TFAP2C | Homo sapiens | Basic helix-span-helix factors (bHSH) |
| MA0525.1 | TP63 | Homo sapiens | p53 domain factors |
| MA0525.2 | TP63 | Homo sapiens | p53 domain factors |
| MA0526.1 | USF2 | Homo sapiens | Basic helix-loop-helix factors (bHLH) |
| MA0526.2 | USF2 | Homo sapiens | Basic helix-loop-helix factors (bHLH) |
| MA0527.1 | ZBTB33 | Homo sapiens | C2H2 zinc finger factors |
| MA0592.1 | ESRRA | Homo sapiens | Nuclear receptors with C4 zinc fingers |
| MA0593.1 | FOXP2 | Homo sapiens | Fork head / winged helix factors |
| MA0595.1 | SREBF1 | Homo sapiens | Basic helix-loop-helix factors (bHLH) |
| MA0596.1 | SREBF2 | Homo sapiens | Basic helix-loop-helix factors (bHLH) |
| MA0598.1 | EHF | Homo sapiens | Tryptophan cluster factors |
| MA0598.2 | EHF | Homo sapiens | Tryptophan cluster factors |
| MA0599.1 | KLF5 | Homo sapiens | C2H2 zinc finger factors |
| MA0600.1 | RFX2 | Homo sapiens | Fork head / winged helix factors |
| MA0600.2 | RFX2 | Homo sapiens | Fork head / winged helix factors |
| MA0620.2 | MITF | Homo sapiens | Basic helix-loop-helix factors (bHLH) |
| MA0630.1 | SHOX | Homo sapiens | Homeo domain factors |
| MA0640.1 | ELF3 | Homo sapiens | Tryptophan cluster factors |
| MA0641.1 | ELF4 | Homo sapiens | Tryptophan cluster factors |
| MA0645.1 | ETV6 | Homo sapiens | Tryptophan cluster factors |
| MA0647.1 | GRHL1 | Homo sapiens | Grainyhead domain factors |
| MA0649.1 | HEY2 | Homo sapiens | Basic helix-loop-helix factors (bHLH) |
| MA0650.1 | HOXA13 | Homo sapiens | Homeo domain factors |
| MA0651.1 | HOXC11 | Homo sapiens | Homeo domain factors |
| MA0652.1 | IRF8 | Homo sapiens | Tryptophan cluster factors |
| MA0653.1 | IRF9 | Homo sapiens | Tryptophan cluster factors |
| MA0655.1 | JDP2 | Homo sapiens | Basic leucine zipper factors (bZIP) |
| MA0656.1 | JDP2(var.2) | Homo sapiens | Basic leucine zipper factors (bZIP) |
| MA0657.1 | KLF13 | Homo sapiens | C2H2 zinc finger factors |
| MA0659.1 | MAFG | Homo sapiens | Basic leucine zipper factors (bZIP) |
| MA0660.1 | MEF2B | Homo sapiens | MADS box factors |
| MA0662.1 | MIXL1 | Homo sapiens | Homeo domain factors |
| MA0665.1 | MSC | Homo sapiens | Basic helix-loop-helix factors (bHLH) |
| MA0667.1 | MYF6 | Homo sapiens | Basic helix-loop-helix factors (bHLH) |
| MA0668.1 | NEUROD2 | Homo sapiens | Basic helix-loop-helix factors (bHLH) |
| MA0672.1 | NKX2-3 | Homo sapiens | Homeo domain factors |
| MA0673.1 | NKX2-8 | Homo sapiens | Homeo domain factors |
| MA0674.1 | NKX6-1 | Homo sapiens | Homeo domain factors |
| MA0678.1 | OLIG2 | Homo sapiens | Basic helix-loop-helix factors (bHLH) |
| MA0680.1 | PAX7 | Homo sapiens | Paired box factors |
| MA0684.1 | RUNX3 | Homo sapiens | Runt domain factors |
| MA0685.1 | SP4 | Homo sapiens | C2H2 zinc finger factors |
| MA0686.1 | SPDEF | Homo sapiens | Tryptophan cluster factors |
| MA0688.1 | TBX2 | Homo sapiens | T-Box factors |
| MA0690.1 | TBX21 | Homo sapiens | T-Box factors |
| MA0692.1 | TFEB | Homo sapiens | Basic helix-loop-helix factors (bHLH) |
| MA0693.2 | VDR | Homo sapiens | Nuclear receptors with C4 zinc fingers |
| MA0700.1 | LHX2 | Homo sapiens | Homeo domain factors |
| MA0711.1 | OTX1 | Homo sapiens | Homeo domain factors |
| MA0712.1 | OTX2 | Homo sapiens | Homeo domain factors |
| MA0714.1 | PITX3 | Homo sapiens | Homeo domain factors |
| MA0717.1 | RAX2 | Homo sapiens | Homeo domain factors |
| MA0718.1 | RAX | Homo sapiens | Homeo domain factors |
| MA0719.1 | RHOXF1 | Homo sapiens | Homeo domain factors |
| MA0727.1 | NR3C2 | Homo sapiens | Nuclear receptors with C4 zinc fingers |
| MA0729.1 | RARA | Homo sapiens | Nuclear receptors with C4 zinc fingers |
| MA0734.1 | GLI2 | Homo sapiens | C2H2 zinc finger factors |
| MA0738.1 | HIC2 | Homo sapiens | C2H2 zinc finger factors |
| MA0745.1 | SNAI2 | Homo sapiens | C2H2 zinc finger factors |
| MA0748.1 | YY2 | Homo sapiens | C2H2 zinc finger factors |
| MA0750.1 | ZBTB7A | Homo sapiens | C2H2 zinc finger factors |
| MA0750.2 | ZBTB7A | Homo sapiens | C2H2 zinc finger factors |
| MA0754.1 | CUX1 | Homo sapiens | Homeo domain factors |
| MA0756.1 | ONECUT2 | Homo sapiens | Homeo domain factors |
| MA0759.1 | ELK3 | Homo sapiens | Tryptophan cluster factors |
| MA0761.1 | ETV1 | Homo sapiens | Tryptophan cluster factors |
| MA0762.1 | ETV2 | Homo sapiens | Tryptophan cluster factors |
| MA0764.1 | ETV4 | Homo sapiens | Tryptophan cluster factors |
| MA0765.1 | ETV5 | Homo sapiens | Tryptophan cluster factors |
| MA0768.1 | LEF1 | Homo sapiens | High-mobility group (HMG) domain factors |
| MA0770.1 | HSF2 | Homo sapiens | Heat shock factors |
| MA0773.1 | MEF2D | Homo sapiens | MADS box factors |
| MA0776.1 | MYBL1 | Homo sapiens | Tryptophan cluster factors |
| MA0777.1 | MYBL2 | Homo sapiens | Tryptophan cluster factors |
| MA0785.1 | POU2F1 | Homo sapiens | Homeo domain factors |
| MA0787.1 | POU3F2 | Homo sapiens | Homeo domain factors |
| MA0795.1 | SMAD3 | Homo sapiens | SMAD/NF-1 DNA-binding domain factors |
| MA0800.1 | EOMES | Homo sapiens | T-Box factors |
| MA0807.1 | TBX5 | Homo sapiens | T-Box factors |
| MA0810.1 | TFAP2A(var.2) | Homo sapiens | Basic helix-span-helix factors (bHSH) |
| MA0814.1 | TFAP2C(var.2) | Homo sapiens | Basic helix-span-helix factors (bHSH) |
| MA0815.1 | TFAP2C(var.3) | Homo sapiens | Basic helix-span-helix factors (bHSH) |
| MA0819.1 | CLOCK | Homo sapiens | Basic helix-loop-helix factors (bHLH) |
| MA0821.1 | HES5 | Homo sapiens | Basic helix-loop-helix factors (bHLH) |
| MA0823.1 | HEY1 | Homo sapiens | Basic helix-loop-helix factors (bHLH) |
| MA0824.1 | ID4 | Homo sapiens | Basic helix-loop-helix factors (bHLH) |
| MA0828.1 | SREBF2(var.2) | Homo sapiens | Basic helix-loop-helix factors (bHLH) |
| MA0830.1 | TCF4 | Homo sapiens | Basic helix-loop-helix factors (bHLH) |
| MA0833.1 | ATF4 | Homo sapiens | Basic leucine zipper factors (bZIP) |
| MA0836.1 | CEBPD | Homo sapiens | Basic leucine zipper factors (bZIP) |
| MA0841.1 | NFE2 | Homo sapiens | Basic leucine zipper factors (bZIP) |
| MA0844.1 | XBP1 | Homo sapiens | Basic leucine zipper factors (bZIP) |
| MA0850.1 | FOXP3 | Homo sapiens | Fork head / winged helix factors |
| MA0852.2 | FOXK1 | Homo sapiens | Fork head / winged helix factors |
| MA0861.1 | TP73 | Homo sapiens | p53 domain factors |
| MA0862.1 | GMEB2 | Homo sapiens | SAND domain factors |
| MA0863.1 | MTF1 | Homo sapiens | C2H2 zinc finger factors |
| MA0866.1 | SOX21 | Homo sapiens | High-mobility group (HMG) domain factors |
| MA0872.1 | TFAP2A(var.3) | Homo sapiens | Basic helix-span-helix factors (bHSH) |
| MA0878.1 | CDX1 | Homo sapiens | Homeo domain factors |
| MA0901.1 | HOXB13 | Homo sapiens | Homeo domain factors |
| MA0902.1 | HOXB2 | Homo sapiens | Homeo domain factors |
| MA0908.1 | HOXD11 | Homo sapiens | Homeo domain factors |
| MA0909.1 | HOXD13 | Homo sapiens | Homeo domain factors |
| MA0914.1 | ISL2 | Homo sapiens | Homeo domain factors |
| MA1100.1 | ASCL1 | Homo sapiens | Basic helix-loop-helix factors (bHLH) |
| MA1101.1 | BACH2 | Homo sapiens | Basic leucine zipper factors (bZIP) |
| MA1102.1 | CTCFL | Homo sapiens | C2H2 zinc finger factors |
| MA1104.1 | GATA6 | Homo sapiens | Other C4 zinc finger-type factors |
| MA1105.1 | GRHL2 | Homo sapiens | Grainyhead domain factors |
| MA1108.1 | MXI1 | Homo sapiens | Basic helix-loop-helix factors (bHLH) |
| MA1109.1 | NEUROD1 | Homo sapiens | Basic helix-loop-helix factors (bHLH) |
| MA1113.1 | PBX2 | Homo sapiens | Homeo domain factors |
| MA1114.1 | PBX3 | Homo sapiens | Homeo domain factors |
| MA1115.1 | POU5F1 | Homo sapiens | Homeo domain factors |
| MA1116.1 | RBPJ | Homo sapiens | Rel homology region (RHR) factors |
| MA1117.1 | RELB | Homo sapiens | Rel homology region (RHR) factors |
| MA1118.1 | SIX1 | Homo sapiens | Homeo domain factors |
| MA1119.1 | SIX2 | Homo sapiens | Homeo domain factors |
| MA1122.1 | TFDP1 | Homo sapiens | Fork head / winged helix factors |
| MA1123.1 | TWIST1 | Homo sapiens | Basic helix-loop-helix factors (bHLH) |
| MA1125.1 | ZNF384 | Homo sapiens | C2H2 zinc finger factors |
| MA1146.1 | NR1A4::RXRA | Homo sapiens | Nuclear receptors with C4 zinc fingers::Nuclear receptors with C4 zinc fingers |
| MA1147.1 | NR4A2::RXRA | Homo sapiens | Nuclear receptors with C4 zinc fingers::Nuclear receptors with C4 zinc fingers |
| MA1148.1 | PPARA::RXRA | Homo sapiens | Nuclear receptors with C4 zinc fingers::Nuclear receptors with C4 zinc fingers |
| MA1149.1 | RARA::RXRG | Homo sapiens | Nuclear receptors with C4 zinc fingers::Nuclear receptors with C4 zinc fingers |
| MA1151.1 | RORC | Homo sapiens | Nuclear receptors with C4 zinc fingers |
| MA1418.1 | IRF3 | Homo sapiens | Tryptophan cluster factors |
| MA1419.1 | IRF4 | Homo sapiens | Tryptophan cluster factors |
| MA1420.1 | IRF5 | Homo sapiens | Tryptophan cluster factors |
