# Supplementary material for: Identification of upstream transcription factor binding sites in orthologous genes using mixed Student’s t-test statistics
Source: PLoS Comput Biol. 2022 Jun 7;18(6):e1009773. doi: 10.1371/journal.pcbi.1009773 (PMC9205514; doi:10.1371/journal.pcbi.1009773)
Supplement: Attachment — Submitted filename: Response-to-reviewers.docx [file pcbi.1009773.s005.docx]

Dear reviewers and editor,

I am pleased to resubmit our original research article entitled "Identification of upstream transcription factor binding sites in orthologous genes using mixed Student’s t-test statistics" for consideration for publication in PLOS Computational Biology.

We want to thank the reviewer and editor for their time and for the constructive comments on our manuscript. We have responded to each comment, and in many cases provided substantial explanation and clarification in the manuscript. We believe this revision has significantly improved our manuscript. Additionally, we have carefully read the manuscript and we have noticed some typos and errors that have been corrected, and imprecise parts have been clarified. To help with the review of the paper, we would like to mention that we have uploaded a new Word document of the manuscript with tracked changes. Please see below our detailed response to your comments. All page numbers refer to the manuscript file with full tracked changes.

Please let me know of your decision at your earliest convenience.

Reviewer #1: Some minor picks:

Line 12: "3.2 Mb/s" - is it mega-base-pairs (Mbp/s)? Please indicate so as to avoid mix up with MB (file size).

Response: We have changed “Mb/s” to “Mbp/s”. Please see line 12 for details.

Line 44: Suggest change "as mentioned below" to "as described below".

Response: We have changed “as mentioned below” to “as described below”. Please see line 44 for details.

Line 54: Suggest change "referred to here as" to "referred here as", deleting "to".

Response: We have deleted “to”. Please see line 54 for details.

The download source code package is missing an essential README file.

Response: We have added a README file to the source code package.

Some major concerns:

(1) This manuscript is well written in general, however, it lack a clear description as how Grit is designed to work: what's the input, what's output, what's the process in which what algorithms was used to perform the motif recognitions calculate and how the reference (Jasper database) was used to make decisions, etc. Figure 1 D looks superficial at this point. It seems necessary to add a section to serve this purpose.

Response: A new section has been added to the manuscript, please see line 87-94 in the manuscript for details. Please also see detailed information as below.

Utilizing the mixed Student’s *t*-test statistics, we developed a tool, called Grit, for screening TFBS by coordinating TFs to their promoter sequences in orthologous genes. The tool takes JASPAR-2020 (specified by the -m option), 2K-set (-i option), and BPS (-b option) as it’s input. Running the tool with default options (-n 10 -z 200 -s 1 -t 0.05 -p 0) will produce a result file (-o option) containing the predicted TFBSs. There are three major steps built into the program: 1) calculate the *RS* for each PWMs presented in each promoter set for orthologous genes using Equation 1; 2) calculate the *p*-values for the significance of *RS* of each genes for each given PWMs using the mixed Student’s *t*-test statistics; and 3) perform multiple testing correction for all *p*-values using the FDR method [25], and retain the TFBSs with FDR ≤ threshold defined by the -t option.

(2) The approaches in this report appear to be innovative in terms of using a new mixed Student t-test to determine the cut off thresholds. The authors compared the outcomes with this tool against a number of other computational tools. However, is it reasonable to suggest that where discrepancies exist between this and other tools, would the TFBS under the spotlights be further verified (e.g. with wet lab methods) in order to make a stronger claim? At the lease this should be a discussion point, I think.

Response: We are sorry that this section was not well written and made the reviewer confused. The Grit – FIMO, Grit – Clover and etc. represented the TFBSs detected by Grit but did not by other tools. The “–” symbol means subtracting. To show the unique features between Grit and the other tools, we investigated the distributions of *CCV* and *SD* for Grit TFBSs and Grit specific TFBSs (TFBSs detected by Grit but did not by other tools, Grit − other). The results indicated that Grit − FIMO and Grit − Swan had significant higher *CCV* values, while Grit − Clover, Grit − Pscan, and Grit − PWMScan had significantly higher *SD* values, than Grit TFBSs (p-value ≤ 0.05, Fig. 3C and 3D).

The result indicated that the Grit specific TFBSs either conserved among species (high *CCV*) or having significant *RS* differences between the target and control sequences (high *SD*). And these are the major differences. Please see line 148-149, and figure legends for Fig. 3 for details.

Would the TFBS under the spotlights be further verified (e.g. with wet lab methods) in order to make a stronger claim? At the lease this should be a discussion point, I think.

Currently, we only assessed Grit with both ChIP-Atlas and ReMap datasets which generated from wet-lab experiment, however, as indicated by the reviewer, further wet-lab is required in the further validation of the TFBSs detected by Grit because they have false positives. We have added this point in the discussion. Please see line 161.

Reviewer #2: In the present manuscript, Huang et al. describe a computational tool ("Grit") that identifies transcription factor binding sites (TFBS) in (alignment-free) upstream DNA sequences of orthologous genes, based on PWMs (positional weight matrices) and a new mixed Student's t-test. The authors tested their tool on simulated data and Chip-Seq datasets from the human genome ChIP-Atlas and compared it with several state-of-the-art TFBS prediction tools. The study is well-written and, in general well-executed, but comes short at some points:

1. It is not entirely clear what the motivation/idea of the new statistical test is. It seems to work on the used simulated and real datasets, but the authors could elaborate more on the theory and model of their mixed Student's t-test.

Response: The motivation of the study is to predict high-scoring TFBS utilizing conservation information among species. The mixed Student’s *t*-test prefers TFBSs either having higher *CCV* or having higher *SD*, or both. We have revised our manuscript, please see line 11, 112-113 for details.

2. In Table 1, the authors state that their tool Grit is species-specific and can identify TFBS conserved among species. This statement is quite ambiguous, and since they only showed results on human datasets, it seems that Grit is human-specific, or at least not tested on other species.

Response: We have changed “conserved among species” to “Utilize conservation information”, sorry for the mistake. Please see line 49 for details.

3. It is not clear what kind of multiple testing approach the authors used (if at all) to correct their calculated p-Values. This is crucial for their statistics and predictions and cannot be omitted!

Response: We perform multiple testing correction for all *p*-values using the FDR method [25], and retain the TFBSs with FDR ≤ threshold defined by the -t option. Please see line 92-94 for details.

4. Which kind of helpful information does Figure 1C provide?
Response: Fig. 1C indicated that the mixed Student’s *t*-test prefers TFBSs either having higher *CCV* or having higher *SD*, or both. Please see line 112-113 for details.

5. In Figure 3B: What is the difference between "TFBS identified by Grit but not by other applications" and "Grit TFBS that do not overlap with other applications"?

Response: “TFBS identified by Grit but not by at least one other applications”, sorry for the mistake. We have revised Fig. 3B.

6. There are several minor typos throughout the manuscript, which should be carefully checked and corrected.

Response: The revised manuscript has been double checked by Editage English editing service.

The authors present an interesting variation of PWMs-based TFBS prediction tools, with an, in general, interesting application of a new statistical model. However, as described in the authors' own words, their tool only "marginally outperforms" current state-of-the-art tools based on one human dataset. To improve their study, the authors should emphasize the details and ideas of their statistical model. They should definitely consider implementing multiple testing corrections and finally apply and test their tool on more datasets.

Response: Thank you very much for your comments, we have changed to “marginally outperforms” to “significantly outperforms” in the revised manuscript. Assessment of Grit with both ChIP-Atlas and ReMap dataset indicated Grit outperformed other tools. Several mistakes in description of the statistical model have been corrected to make it clearer. Test of the Grit tool on more species datasets are undertaken at this time at our laboratory, it’s time consuming and the result will be provided on the Grit web site when finished.
